# Contrasting the co-variability of daytime cloud and precipitation over tropical land and ocean

**DOI:** 10.5194/acp-18-3065-2018

**Published:** 2018-03-02

**Authors:** Daeho Jin, Lazaros Oreopoulos, Dongmin Lee, Nayeong Cho, Jackson Tan

**Affiliations:** 1 University Space Research Association, Columbia, MD, USA; 2 NASA Goddard Space Flight Center, Greenbelt, MD, USA; 3 Morgan State University, Baltimore MD, USA

## Abstract

The co-variability of cloud and precipitation in the extended tropics (35°N–35°S) is investigated using contemporaneous data sets for a 13-year period. The goal is to quantify potential relationships between cloud type fractions and precipitation events of particular strength. Particular attention is paid to whether the relationships exhibit different characteristics over tropical land and ocean. A primary analysis metric is the correlation coefficient between fractions of individual cloud types and frequencies within precipitation histogram bins that have been matched in time and space. The cloud type fractions are derived from Moderate Resolution Imaging Spectroradiometer (MODIS) joint histograms of cloud top pressure and cloud optical thickness in 1°grid cells, and the precipitation frequencies come from the Tropical Rainfall Measuring Mission (TRMM) Multi-satellite Precipitation Analysis (TMPA) data set aggregated to the same grid.

It is found that the strongest coupling (positive correlation) between clouds and precipitation occurs over ocean for cumulonimbus clouds and the heaviest rainfall. While the same cloud type and rainfall bin are also best correlated over land compared to other combinations, the correlation magnitude is weaker than over ocean. The difference is attributed to the greater size of convective systems over ocean. It is also found that both over ocean and land the anti-correlation of strong precipitation with “weak” (i.e., thin and/or low) cloud types is of greater absolute strength than positive correlations between weak cloud types and weak precipitation. Cloud type co-occurrence relationships explain some of the cloud–precipitation anti-correlations. Weak correlations between weaker rainfall and clouds indicate poor predictability for precipitation when cloud types are known, and this is even more true over land than over ocean.

## 1 Introduction

Attempts to estimate precipitation from cloud observations have a long history dating back to the era of first passive thermal infrared observations of clouds (e.g., Richards and Arkin, 1981). Enlisting numerical models to help with the interpretation of observations has not been as helpful as hoped since these models generally do not produce coherent relationships between clouds and precipitation (e.g., Stephens et al., 2010; Gianotti et al., 2012; Jiang et al., 2015), with even cloud-resolving models explicitly representing precipitation processes facing challenges in that respect (e.g., Kooperman et al., 2016; Matsui et al., 2016). In the case of atmospheric global circulation models (AGCMs), it is nearly impossible to resolve individual precipitating processes due to the sub-grid nature of the problem and the excessive computational burden. Hence, for AGCM evaluation, and also for observation-based water budget studies, a synoptic approach for identifying the relationships between cloud and precipitation has been deemed an inevitable compromise.

One example of employing a synoptic approach is the use of the concept of a “cloud regime” (CR) also known as “weather state” (WS; Jakob and Tselioudis, 2003; Rossow et al., 2005; Oreopoulos and Rossow, 2011; Tselioudis et al., 2013; Oreopoulos et al., 2014, 2016) to study precipitation characteristics. Cloud regimes represent the dominant mixtures of cloud types, and can be used as a framework to categorize cloud data in a grid (e.g., Level-3 satellite products). Using the International Satellite Cloud Climatology Project (ISCCP) WSs defined in the extended tropics (35°S–35°N), Lee et al. (2013) provided a comprehensive picture of precipitation characteristics for each WS, with an additional focus on the relationship between the most convective regime (WS1) and precipitation. Rossow et al. (2013) also conducted similar analysis but for precipitation extremes using ISCCP WSs for the deep tropical zone of 15°S–15°N. While such CR-based approaches provide valuable information about the cloud–precipitation relationship at large scales, the precipitation composites by CR encompass large spreads which obscure details of the relationship. Since CRs contain mixtures of clouds types by design, and therefore contain considerable cloud variability, ambiguities in the cloud-precipitation relationships are hard to resolve.

Cloud-precipitation relationships can, however, be examined at a more detailed level with coincident precipitation profile and cloud measurements. An example of this is the “cloud and precipitation feature database” of Liu et al. (2008). The database was derived from observations by the precipitation radar (PR), the Tropical Rainfall Measuring Mission (TRMM) Microwave Imager (TMI), the Visible and Infrared Scanner (VIRS), and the Lightning Imaging System (LIS) aboard the TRMM satellite. The authors performed several case studies with this data set that contrasted continental and oceanic precipitating cloud systems, and found that oceanic storms were generally horizontally larger at 2 km altitudes, but continental storms tended to be vertically more coherent, with a higher top and more severe rainfall. Houze et al. (2015) also reported similar results using solely vertical rainfall profiles from the TRMM PR. While these studies provided a more detailed look at the cloud-precipitation relationship thanks to the high resolution of the TRMM PR (4–5 km footprint at nadir), the penalty was narrow horizontal coverage (swath widths of 215 km before orbit boost and 247 km after orbit boost).

Our study aims to go beyond widely known cloud- precipitation associations (such as geometrically deep and optically thick clouds producing stronger rainfall), and to examine instead more carefully the details of the connections between clouds and precipitation for situations that also include non-heavy precipitation. We thus strive for generality of results by covering the entire tropics and for overcoming the ambiguity of CR-based studies by taking advantage of the ability to break down individual grid-box cloud fractions with the aid of joint cloud histograms. Hence, our paper revisits and explores anew the mesoscale cloud- precipitation relationship via the synoptic approach by employing a Moderate Resolution Imaging Spectroradiometer (MODIS) gridded cloud data set (King et al., 2003; Platnick et al., 2003) and the TRMM Multi-satellite Precipitation Analysis (TMPA) data set (Huffman et al., 2007, 2010). While the MODIS Level-3 data are provided at 1°×1°resolution, the 2-D joint histogram of cloud optical thickness × (*τ*) and cloud top pressure (*p*_*c*_) contains pixel-level cloud information which can be combined with the sub-grid variability of precipitation at the 1°×1°scale, available by virtue of the finer 0.25°×0.25° spatial resolution of TMPA. While still coarser than the TRMM PR data set, the combined MODIS and TMPA data set covers the entire tropics every single day, allowing better generalization of the daytime relationship between clouds and precipitation. We seek to answer questions such as the following: what are the general expectations and limitations in predicting precipitation given a cloud type in the extended tropics? Is there a closer relationship between certain precipitation rates and cloud types? Do answers to the above questions differ substantially between oceans and continents?

The next section introduces the concept of “precipitation histogram” and how it can be matched and correlated to sub-grid cloud type fractions at the grid level. A comprehensive examination and interpretation of cloud and precipitation co-variability over tropical land and ocean follows in Sect. 3. In addition to summarizing the results, the concluding Sect. 4 calls attention to the new insights that emerge from this study and challenges that remain to be addressed about the nature of cloud-precipitation coupling.

## 2 Data and methodology

### 2.1 Cloud and precipitation data

Our passive cloud retrievals come from the Moderate Resolution Imaging Spectroradiometer (MODIS) instrument aboard the Terra and Aqua satellites. The MODIS cloud data set (MOD08_D3 and MYD08_D3; King et al., 2003; Platnick et al., 2003) provides Level-3 cloud products at daily timescales with 1°× 1°horizontal resolution. Among various cloud products, we focus on the 2-D joint histogram of cloud optical thickness (*τ*) and cloud top pressure (*p*_*c*_). The histogram is composed of cloud fraction (CF) values along seven classes of *p*_*c*_ and six classes of *τ* (for a total 42 histogram bins), and contains pixel-level cloud variability information at the 1°scale. The most recent version of the MODIS atmospheric data sets, known as “Collection 6” (Platnick et al., 2017), provides a separate histogram for “partially cloudy” (PCL) pixels, flagged as such by the so-called “clear-sky restoral” algorithm (Pincus et al., 2012; Zhang and Platnick, 2011). The PCL pixels usually represent cloud edge pixels for which the cloud property retrievals are deemed more uncertain (Cho et al., 2015). We opted to include PCL pixels in our analysis by adding the PCL histogram to the nominal histogram because, by doing so, the MODIS cloud climatology becomes more consistent (see Oreopoulos et al., 2014) to that by ISCCP (Rossow and Schiffer, 1991, 1999), which has a long track record in cloud research and can potentially be used in a study similar to this one. In this study, the joint histogram bins are coarsened from 42 bins to nine cloud types because of practical considerations (see Sect. 2.3) as well as our desire to draw an analogy with the ISCCP cloud types (Chen et al., 2000; Rossow and Schiffer, 1999).

The precipitation data set used in our study is the 3B42 research product (version 7) of Tropical Rainfall Measuring Mission (TRMM) Multi-satellite Precipitation Analysis (TMPA; Huffman et al., 2007, 2010; Huffman and Bolvin, 2015). The TMPA pursues the “best” satellite precipitation estimates using TRMM Microwave Imager (TMI) and Precipitation Radar (PR) data as calibrators in merging measurements from several microwave and infrared sensors, and monthly gauge data (over land) from the Global Precipitation Climatology Centre (GPCC; Huffman et al., 2007). The horizontal resolution of TMPA is 0.25°×0.25°covering 50°S to 50°N. TMPA is available from January 1998 with 3-hourly resolution, but we use only the period from December 2002 to November 2015 which overlaps temporally with Aqua and Terra MODIS data. Since we pursue the co-variability of cloud and precipitation, and one of the essential pieces of cloud information is the optical thickness which is only available during daytime, our study relies on measurements only around the Terra and Aqua overpasses of 10:30 and 13:30 local solar time (LST), respectively. We restrict our study to the extended tropical region (35°N–35°S) to avoid ambiguities in the interpretation of the MODIS joint histograms which include progressively more temporal variability towards higher latitudes as data from successive spatially overlapping orbits fall within the same 1° ×1°grid cell. Still, we should note that when various aspects of the analysis were tested on the full TMPA spatial coverage (50°N–50°S), the results were not substantially different. Lastly, since it is well established that precipitation properties over land and ocean are quite different (e.g., Williams and Stanfill, 2002; Zipser et al., 2006; Matsui et al., 2016), we maintain via the MODIS land-water mask (Carroll et al., 2009) distinct land and ocean results throughout our analysis. At the 1°×1°resolution, a grid cell is marked as ocean when the water mask area is greater than 90 %, while it is marked as land when the water mask area is smaller than 10 %. For our extended tropics domain this definition assigns 71.1 % of the grid cells to the ocean and 24.1 % to the land category.

The quality of the TMPA product differs between land and ocean, mainly due to two factors: (1) gauge adjustment which reduces systematic biases in land precipitation and (2) satellite retrieval algorithm differences which result in lower random errors over ocean (Liu, 2016; Sapiano and Arkin, 2009; Tian and Peters-Lidard, 2010). We assert that our findings about ocean-land differences are not much affected by these algorithm differences because, first, random errors should be suppressed due to large sample size and, second, our analysis is largely based on deviations from the mean state. Nevertheless, it is understood that TMPA overall performs less reliably in certain situations such as continental warm rains (Kidder and Vonder Haar, 1995; Kummerow et al., 2015).

### 2.2 Matching precipitation data to cloud grid

**Figure 1.**
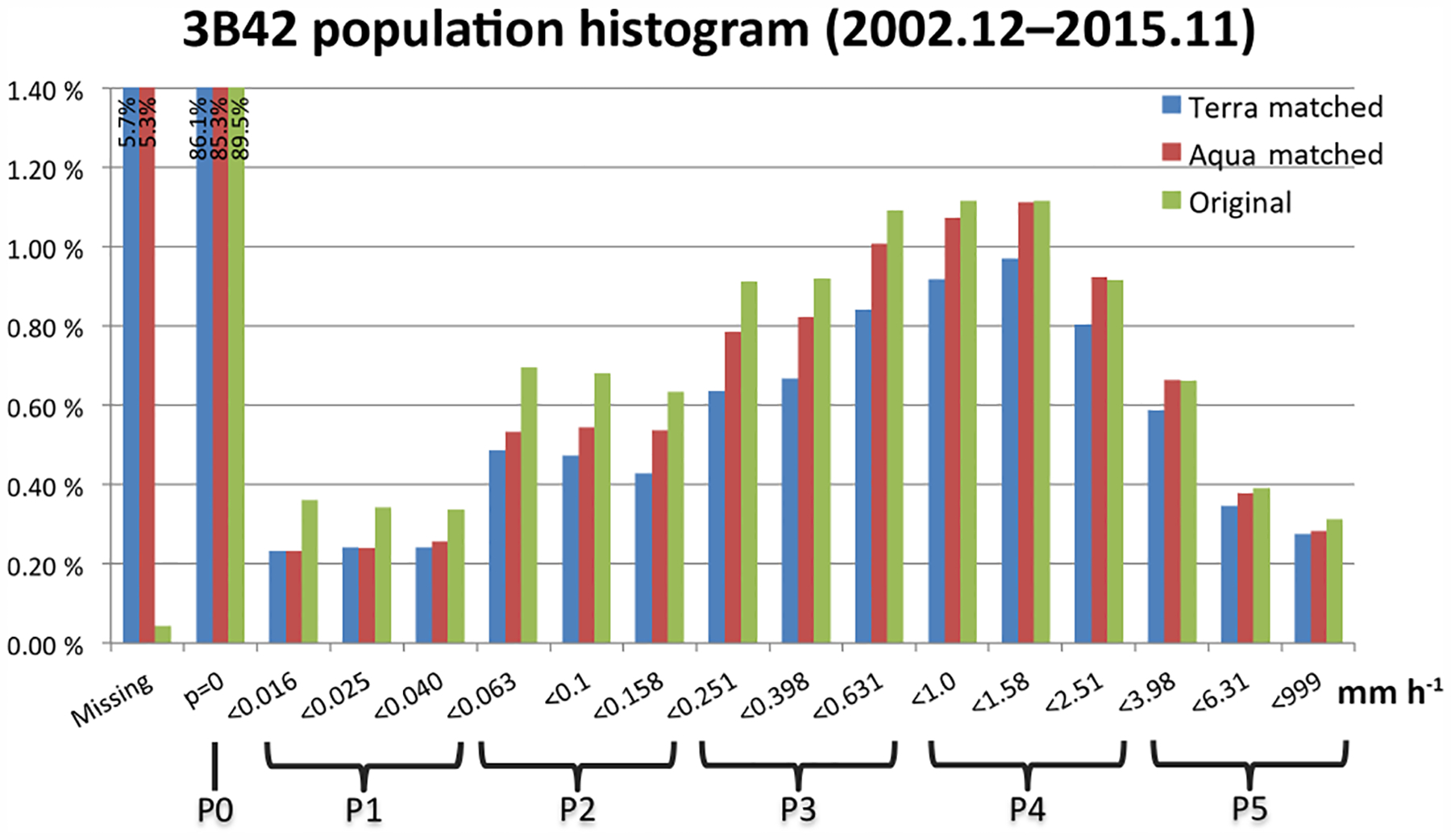
Histograms of TMPA original 0.25°0*×*25°3-hourly 3B42 precipitation data (green), and subsets matched with daytime Terra (blue) and Aqua (red), from December 2002 to November 2015 in the extended tropics domain. The boundaries that define the six simplified precipitation groups are shown at the bottom.

Because the 3B42 data set has higher spatial resolution than the MODIS Level-3 cloud data set, we resample it to the 1° ×1°resolution of the MODIS data set. Previous studies averaged precipitation rates to a single value representing grid mean (e.g., Lee et al., 2013; Rossow et al., 2013). In this study, a marginal histogram of 3B42 0.25° ×0.25°grid precipitation rates is created for each 1° ×1°grid cell. The idea of such 1°×1°precipitation histograms was drawn from our other main data set, the MODIS joint 2-D histogram of *p*_*c*_ −*τ*, which preserves a certain degree of sub-grid cloud information (although not of the actual spatial distribution of the sub-grid variability). So, in a sense, sub-grid information about precipitation rate can also be preserved in the form of a histogram by assigning the 16 values (when there are no missing values) of precipitation rate at 0.25°×0.25°resolution to pre-defined bins to create a marginal histogram at 1° ×1°grid cell. The histogram is normalized by dividing each bin count by the total count in the histogram bins, i.e., 16, in the default case of no missing value. Hence, each bin value falls between 0 and 1 in multiples of 1 / 16, and sub-grid precipitation rates are interpreted as areal fractions of specific ranges of precipitation rates. Of the 16 precipitation histogram bins, 1 corresponds to “no-rain” and the remaining 15 bins correspond to rain rates greater than 0. Histogram bin boundaries are selected with 15 logarithmically spaced intervals to ensure a more even distribution of counts (see Fig. 1). Figure 1 shows the distribution of precipitation rate of the original TMPA data in our extended tropics domain according to this histogram binning approach. We see that the amount of missing data is negligible, and that the “no-rain” bin has an 89.5 % share of all data points. The rain rate around 1 mmh^−1^ has a maximum share near 1.1 %, and extreme values are below 0.4 % at both low and high rain rates.

In addition to the trivial matching of grid cells, the TMPA and MODIS observations also need to be matched in time. Since MODIS Level-3 cloud data come from the aggregation of retrieved satellite observation along the Terra or Aqua paths, and since these satellites are in a Sun-synchronous orbit, each grid cell of a daily MODIS map has a limited range of nominal LST, but has a varying Coordinated Universal Time (UTC), the time-keeping system of TMPA. The UTC of each grid cell can be estimated from the mean solar zenith angle (SZA) available as a MODIS Level-3 variable, and the latitude and time information for each grid cell. Because of minimal overlap of satellite orbits in the tropics, the mean SZA value is a result of mostly (small) spatial variations within the 1°×1°grid cell. After identifying the UTC corresponding to the grid cell of cloud data, the proper TMPA data points can be extracted. Since the TMPA data are available at 3 h intervals, TMPA data centered, say, at 12:00 LTC, will be matched with MODIS data between 10:30 and 13:30 LTC.

The histograms of TMPA tropical rainfall rate that match Terra and Aqua paths spatially and temporally are also shown in Fig. 1. One notable change from the original TMPA data to Terra- or Aqua-matched data is that the portion of missing data now surges to over 5 % of total data points. Most of these missing data are traced back to unavailable Level-3 MODIS data, for reasons such as absence of clouds or gaps between consecutive Terra-Aqua orbits at low latitudes. Other differences in occurrence frequencies between original and matched data are probably due to the diurnal cycle of precipitation. At the Terra overpass time of around 10:30 LST, precipitation is relatively weak over both land and ocean (e.g., Yang and Smith, 2006; Kikuchi and Wang, 2008). This appears in Fig. 1 as Terra-matched precipitation having smaller frequencies than the original and the Aqua-matched precipitation, although it is somewhat improper to directly compare Terra- or Aqua-matched data with fully sampled data because the higher ratio of available (non-missing) data in the fully sampled data propagates as higher relative frequency in the various precipitation bins. It is also notable that for weak-to-moderate precipitation rates (less than 1 mmh^−1^), even Aqua-matched precipitation is (slightly) lower in percentage terms than fully sampled TMPA precipitation, which can be interpreted as weak-to-moderate precipitation being more frequent outside the time windows of Terra and Aqua overpasses.

### 2.3 Analysis method and simplification of cloud and precipitation histograms

The simplest and most straightforward method to measure the co-variability of two variables is to calculate their cross-correlation coefficients, namely Pearson’s *r*. In this study, the cloud fraction values in each bin of the *p*_*c*_–*τ* joint histogram and the relative frequencies in the precipitation histogram form large arrays (O(1 000 000)) in the spatiotemporal domain, from which we can calculate correlation coefficients as time and location varies. The original resolution of the *p*_*c*_ *–τ* and precipitation histograms yields 672 (= 42 CF bins×16 precipitation bins) correlation coefficients. Analysis and visualization of such a large number of coefficients are impractical, hence we pursue an analysis where both the cloud and precipitation histograms are coarsened.

**Figure 2.**
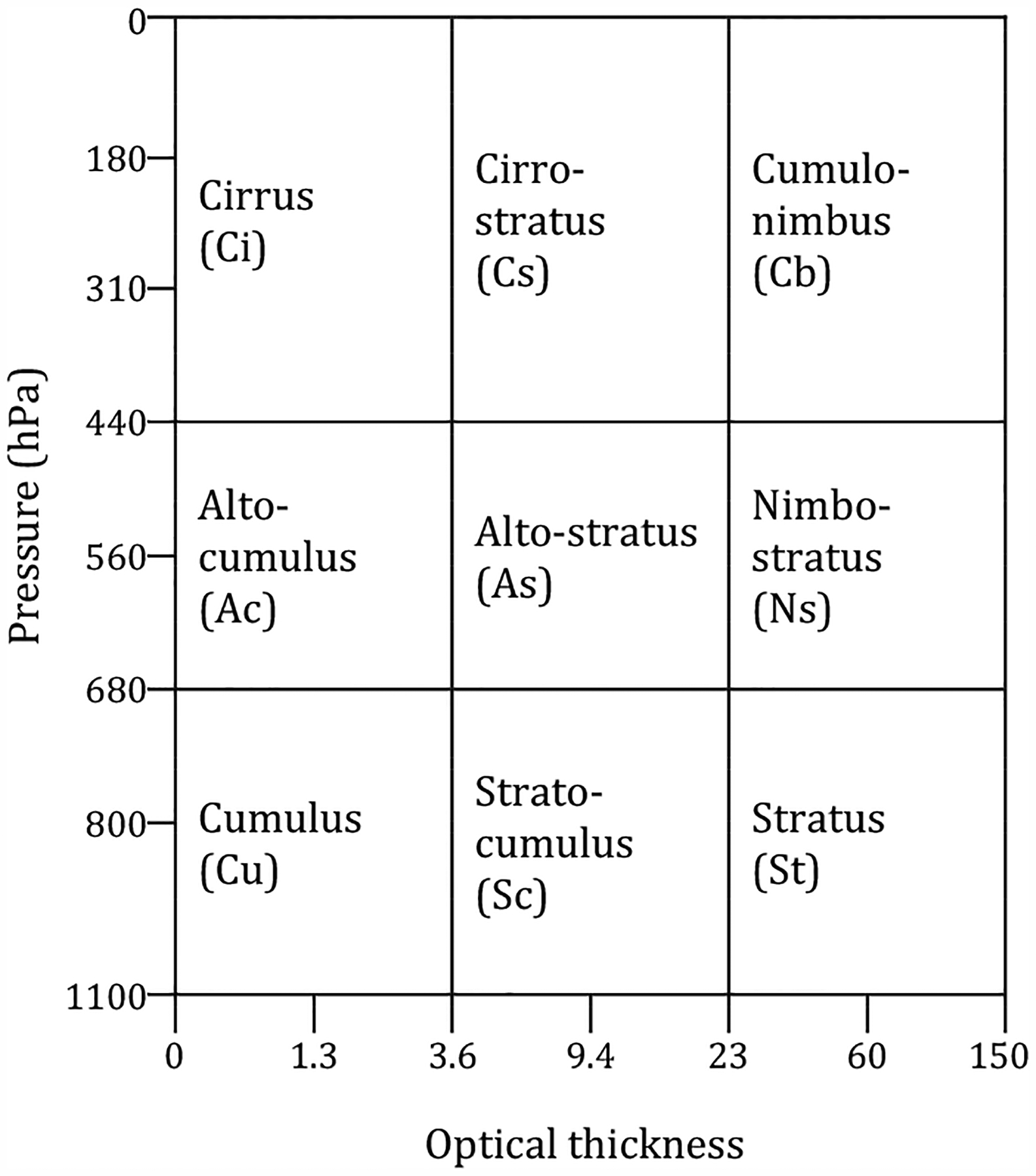
ISCCP cloud types assigned to groups of bins in MODIS joint histogram of *τ*–*p*_*c*_.

Reducing the 42 bins of the cloud histogram allows us to make a more intuitive physical connection with the nine standard ISCCP cloud types of Rossow and Schiffer (1999). While these cloud types were given the same names as the standard cloud types seen by human observers from the ground and have some affinity with them, they are only loosely connected with the widely recognized traditional cloud types. Figure 2 shows the *p*_*c*_ and *τ* range for each cloud type. Low and mid-level cloud types are composed of four CF bins (= two *p*_*c*_ classes × two *τ* classes) while high cloud types are composed of six CF bins (= three *p*_*c*_ classes × two *τ* classes). Hence, the CF value of each cloud type comes from the summation of either four or six CF bin values of the original 2-D joint histogram.

Similarly, the 16 histogram bins of precipitation are reduced to six groups. The “no-rain” bin is unchanged, and the other 15 bins of measurable rainfall are resampled to five precipitation groups (each referred to as a “P-group” hereafter) by summing three consecutive precipitation bins, as shown at the bottom of Fig. 1. Each P-group is labeled from P1 to P5, with P1 representing the lightest precipitation, and P5 representing the heaviest precipitation. For simplicity, the same symbols are henceforth also used to represent the frequency of occurrence within these groups, since their meaning is always clear by the context.

Our histogram coarsening reduces the number of correlation coefficients to 54 (= nine cloud type CF values six × P-group frequencies). Since the Terra and Aqua data (and matched precipitation data) are considered as a single ensemble, our results represent the local cloud-precipitation co-variability for the 6 h daytime period spanning 1.5 h before the Terra overpass to 1.5 h after the Aqua overpass.

## 3 Land–ocean difference of cloud–precipitation relationships

### 3.1 Basic statistics and composite means of cloud and precipitation data

**Figure 3.**
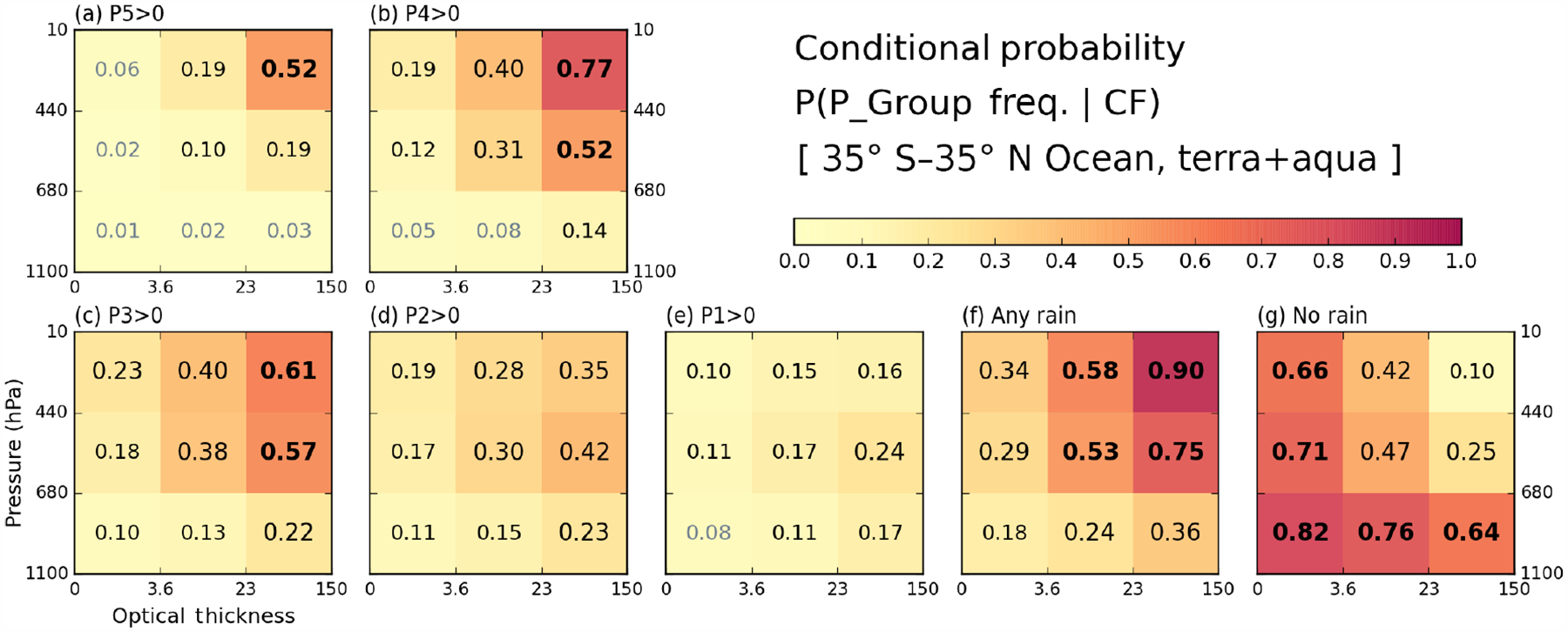
(a–e) Conditional probabilities of precipitation within a P-group (from TMPA) given occurrences of a cloud type (from MODIS) over ocean in the extended tropics from December 2002 to November 2015. (f) Conditional probabilities of any rain amount (sum of all P-group frequencies). (g) Conditional probabilities of no rain co-occurring with cloud. The CF threshold for cloud type occurrence is 6.25 %.

**Figure 4.**
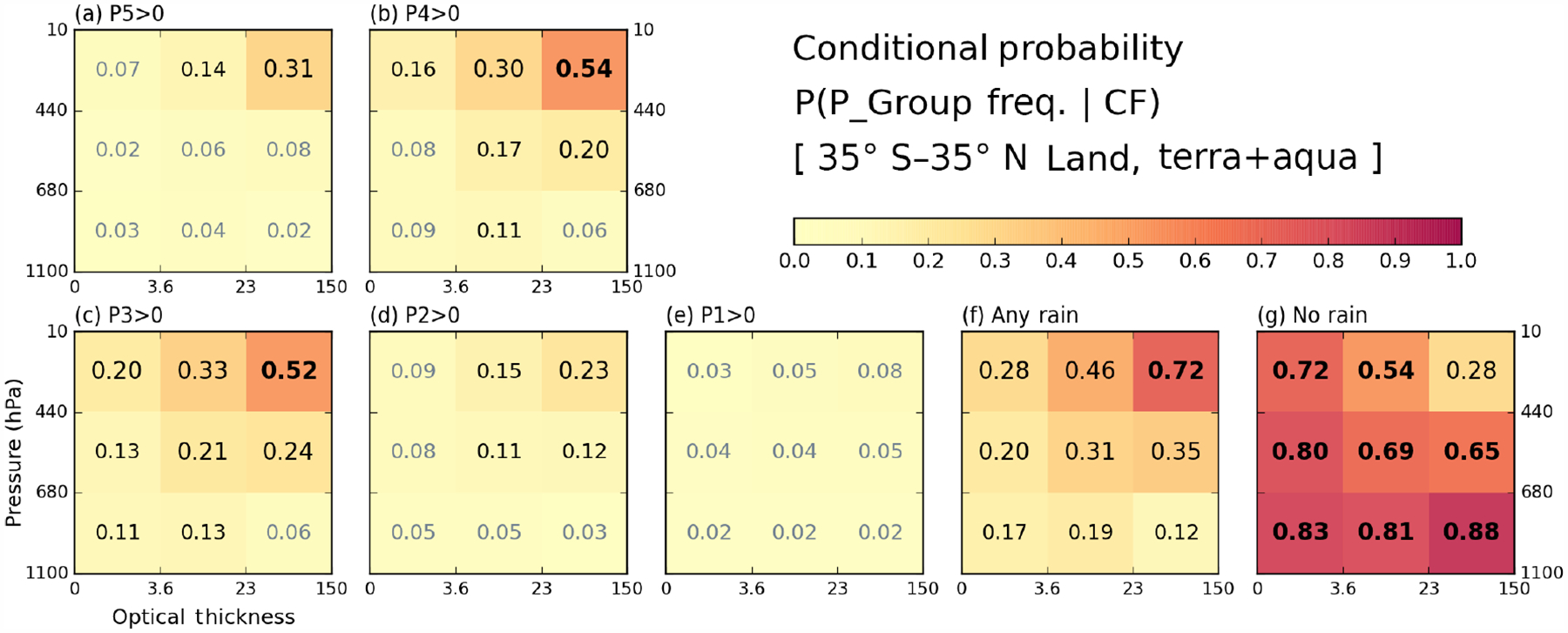
Same as Fig. 3, but over land.

Before examining correlations between cloud and precipitation data, it is illuminating to examine the basic statistical information and mean states of both histograms from which correlations are extracted. First, we examine the P-groups that co-exist with certain cloud type fractions at the grid level. Figures 3 and 4 show the conditional probability of P-group occurrence under the condition that a particular cloud type exists over ocean (Fig. 3) and land (Fig. 4). For example, for all oceanic 1°× 1°grid cells with cumulonim bus (*Cb*) clouds occurring, about 52 % of the grid cells report P5 precipitation at one or more 0.25°sub-grid cell(s) (Fig. 3a, upper-right bin). The threshold CF that determines cloud occurrence is set to 6.25 %, i.e., the same threshold fraction (1*/*16) that defines precipitation occurrence. We note that P-groups are not mutually exclusive because several P-groups can occur simultaneously in a 1°×1°grid cell.

**Figure 5.**
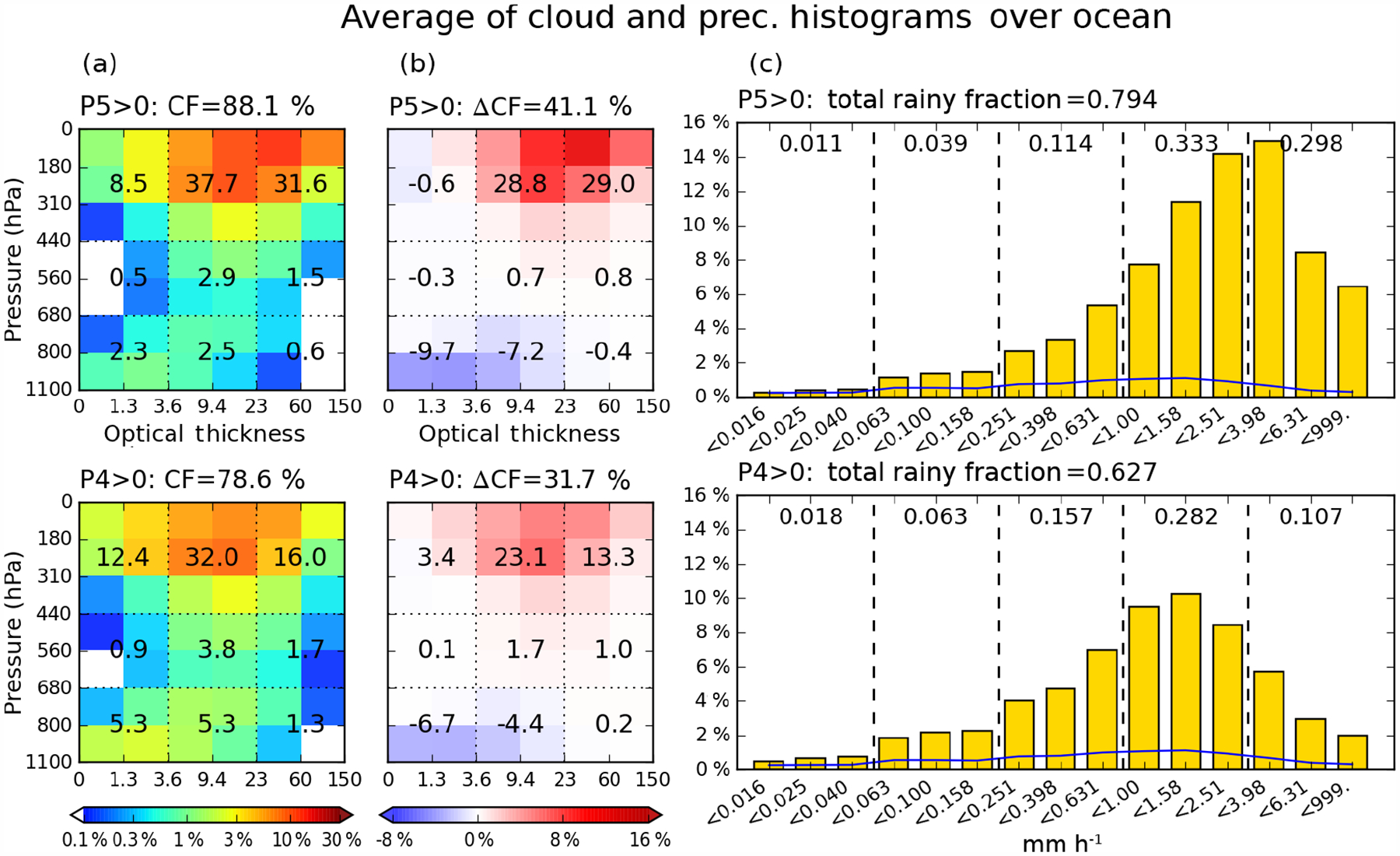
Conditional composite mean of 2-D joint histogram of *p*_*c*_ and *τ* (left column), differences from overall (unconditional) mean (middle column), and precipitation histogram (right column) over the extended tropical oceans for 13 years. Top row is for P5, while bottom row is for P4 precipitation. Blue lines in precipitation histograms indicate the overall mean. Both cloud and precipitation overall means correspond to the entire domain, and not just ocean. Numbers on cloud histograms are the cloud fraction (CF; in percentage) of each cloud type, which is the sum of four or six histogram bin values assigned to the cloud type. The sum of all values is equal to the total cloud fraction provided above each panel. Numbers on precipitation histograms are the fraction of each P-group, P1 (left) to P5 (right), obtained as the sum of three individual bin values. Total rainy fraction is the sum of all P-groups’ fractions (i.e., sum of 15 individual bin values).

**Figure 6.**
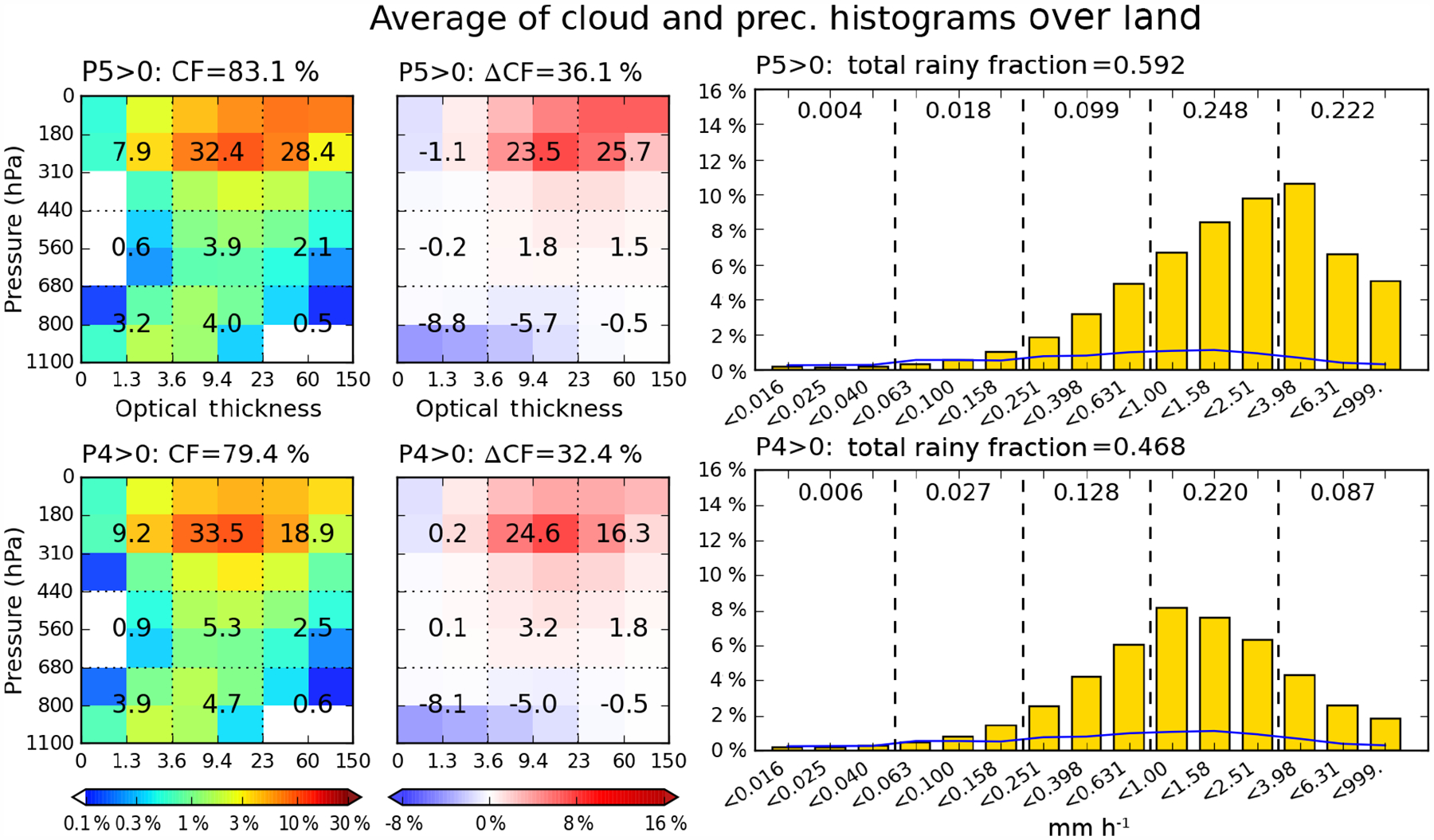
Same as Fig. 5, but over land.

Over ocean, the cloud type co-occurring the most with precipitation rates of moderate to heavy intensity is, not surprisingly, *Cb*. The P-group most likely to occur alongside *Cb* clouds is P4 with a probability of 0.77 (Fig. 3b). The probability of P5 group occurrence is lower at 0.52 but also comes with an overall P5 population smaller than that of P4 (Fig. 1 and Table 1). When precipitation of any intensity is considered (Fig. 3f), besides *Cb* having the highest probability of precipitation, 0.90, oceanic nimbostratus (*Ns*) also emerges with a high probability of 0.75. The no-rain occurrences are, not surprisingly, more strongly associated with thin and/or low clouds (so-called “weak” clouds), topped by the 0.82 probability for cumulus (*Cu*) clouds. It is notable that no-rain probabilities are clearly distinguishable from those of the weak P1 or P2 rain groups not only by the probability of these P-groups occurring (we note that the population of the no-rain case is much larger) but also by how the probability varies with cloud type within the precipitation group (e.g., compare *Cu* and *Ns* in Fig. 3e and g as an extreme contrast). Comparing Figs. 3 and 4, we see that land clouds generally have a smaller chance of precipitation co-existing with clouds at the 1°scale. Even the P4 precipitation probability of *Cb* clouds is only 0.54 (Fig. 4b), far lower than its oceanic counterpart of 0.77. For the case of rainfall with any intensity (Fig. 4f), the precipitation probability of *Ns* is only 0.35 compared to 0.75 over ocean. The precipitation probability of mid-level altostratus (*As*) also decreases from 0.53 to 0.31, so mid-level clouds seem to be particularly less active precipitation producers over land. In addition, the lightest rain group, P1, over land is not associated with any particular cloud type (Fig. 4e vs. Fig. 3e), while the no-rain case exhibits strong probability dependence on cloud type. The issue of less rain over land is also covered in the next composite plots (Figs. 5 and 6).

Figures 5 (ocean) and 6 (land) show composite mean cloud and precipitation histograms, for occurrences of the strongest precipitation groups P5 and P4 (i.e., at least one of the sub-grids within the 1°× 1°grid cell has a precipitation rate belonging to the P5 or P4 group). When P5 occurs over ocean (Fig. 5), both cloud and rainy fractions exceed those of the P4 cases. On the cloud side, *Cb* exhibits the largest increases in CF when moving from the P4 to the P5 composite. For the P5 composite, the largest CFs (red color) are located in the bins with *p*_*c*_ below 310 hPa and the *τ* bins extending from 9.4 to 60, while in the P4 composite, CF peaks in the bin bounded by 310 and 180 hPa, and with *τ* between 3.6 and 23. Conversely, thin (*t <* 3.6) cloud CFs as well as stratocumulus (*S*c) CF are smaller in the P5 composite than the P4 composite. However, it cannot be determined from this analysis alone whether the increased amounts of thin and *Sc* clouds in the P4 composite are directly linked with the occurrence of P4 precipitation, or if they are a consequence of increased chance of co-existence with other clouds producing P4 precipitation. The CFs of mid-level clouds increase only slightly from P5 to P4 composites in terms of absolute values, but these increases are quite large in a relative sense because absolute CF values for these clouds are very small in the MODIS climatology.

Consistent with the CF changes, the total rainy fraction, defined as the sum of the 15 precipitation histogram bin frequencies excluding the “no-rain” bin in 1°×1°grid cells, also increases in the P5 composite (0.794 vs. 0.627). The mean precipitation histogram in the P5 composite (Fig. 5 top right) exhibits a peak within the P5 group, but the fraction of total precipitation in the P4 group is larger. This does not come as a surprise because, first, the absolute population of P4 is higher than that of P5 (Fig. 1), and second, most P5 precipitation events co-occur with P4 precipitation events at 1°× 1°resolution (Table 1). The P4 fractional contribution in the P5 composite is also larger than the P4 contribution in the P4 composite (Fig. 5 bottom right), while the light-to-moderate P-group (P1–P3) fractions are slightly larger in the P4 composite compared to the P5 composite. This indicates that stronger precipitation events also have a weaker tail towards lower rainfall rates. In terms of total rainy fraction, considering that approximately 38 % of the P4 composite population overlaps with the P5 composite (Table 1), we see that the spatial extents of oceanic rain systems producing P4 but not P5 are often much smaller than systems producing P5.

We also examined the geographical distributions of P4 and P5 occurrence frequency (Fig. S1 in the Supplement), and found that the distribution maps look very similar, with the regions significantly skewed towards one of P4 or P5 being very few. This result suggests that the P4 and P5 composites in Fig. 5 are related and likely respectively capture the developing and mature stage of mesoscale convective systems (MCSs). The review of Houze (2004) and Chapter 9 of Houze (2014) describe MCS as the combined system of a large region of stratiform precipitation paired with individual or clustered *Cb* clouds, yielding thus a variety of cloud and precipitation structures (Houze et al., 1990). The P5 composite patterns of cloud and precipitation shown in Fig. 5 are in accordance with such MCS characteristics, i.e., strong convective clouds and a broad spectrum of precipitation.

Figure 6 shows the same P4 and P5 composite means as Fig. 5, but over land. Comparing the top and bottom panels of Fig. 6, we see that the general characteristics of the differences between P4 and P5 land composites are similar to their oceanic counterparts. For example, the total CF and rainy fraction increase from the P4 to the P5 composites, accompanied by larger CFs of *Cb* clouds, and P4 group fractional contribution in the P5 composite. However, there are also notable differences, such as total CF difference between P4 and P5 composites being smaller over land than over ocean. Over land, smaller CFs can produce P5-magnitude precipitation while larger CFs are needed for P4-magnitude precipitation compared to ocean. The total rainy fractions of P4 and P5 composites are also smaller over land. For example, when P5 occurs, 79 % of oceanic sub-grids at 1°scales are precipitating, while the same is true for only 59 % of continental sub-grids. For P4 composites, the values are 63 % over ocean vs. 47 % over land. These are strong indications that continental systems producing heavy rainfall are in general smaller in size than their oceanic counterparts (Liu et al., 2008; Houze, 2014, Chapter 9; Houze et al., 2015).

**Figure 7.**
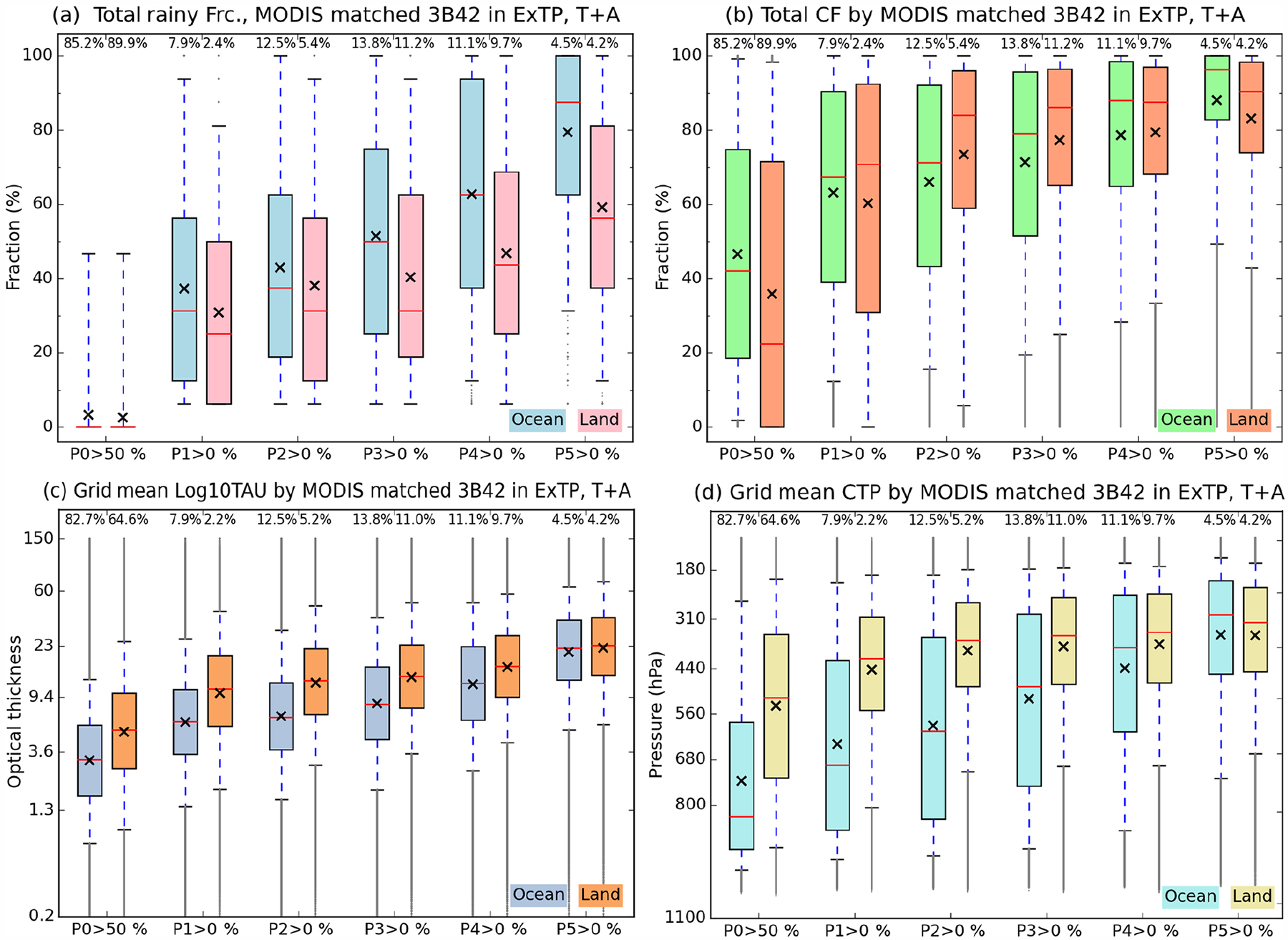
Box-and-whisker plot of **(a)** the total rainy fraction, **(b)** the total cloud fraction, **(c)** the grid-mean log10*(τ)*, and (d) the grid-mean *p*_*c*_ conditioned by precipitation groups, separately for ocean and land. The median values are shown as red horizontal lines, and the mean values are shown as black crosses. The vertical width of the boxes indicates the interquartile range (25th–75th percentile), and the whiskers extend from 5 to 95 % values. Percentage numbers above the boxes indicate the occurrence ratio of each P-group relative to the total ocean or land grid cells.

The distributions of total rainy fraction as well as grid mean cloud properties by P-group are further examined in Fig. 7, which shows box plots of total rainy fraction, CF, log10(τ), and *p*_*c*_ distributions. Over ocean, both total rainy fraction and CF generally increase monotonically with precipitation. However, the picture is somewhat different for land. From P2 to P5, both the median and mean values of land CF are quite similar (Fig. 7b). As a result, in the P2 case, the land CF median is nearly 10 % greater than the ocean CF median, while it becomes 5 % smaller than its ocean CF counterpart in the case of P5. At the same time, the total rainy fraction over land appears to monotonically increase in the same way as over ocean, albeit with a notably smaller absolute value and slope of growth. Hence, it appears that over land, similar amounts of CF (e.g., 70 to 80 %) in a 1°×1° grid cell are involved in a broad range of precipitation rates, while the fraction of raining clouds in the grid cell is much smaller compared to ocean. Collectively, these results indicate reduced predictability of precipitation from knowledge of CF over land, at least with the precipitation data set at hand.

Houze (2014, Chapter 9) and Houze et al. (2015) noted that shallow and isolated clouds producing “warm rain” are mostly oceanic phenomena, while the size of MCSs is generally larger over ocean than land. These two different precipitating sources can explain the big contrast over ocean of total rainy fraction between P2 (median 35 %) and P5 (median 85 %); over land the difference is less than 30 %. In addition, Fig. 7c and d show that light-to-moderate precipitation groups (P1–P3) over ocean are associated with optically thinner and shallower clouds, more evidence of the prevalence of marine “warm” rain processes. Note also the larger variability (taller interquartile box) of oceanic *p*_*c*_ for light precipitation compared to heavy precipitation, indicating that the former is harder to relate to particular cloud types. For continental light precipitation, associated clouds are optically thicker and of higher altitude than oceanic counterparts, but TMPA’s potential weakness in identifying such precipitation, as described in Sect. 2.1, may be affecting the land results of Fig. 7.

In summary, the 1°× 1°spatiotemporally matched cloud and precipitation data suggest that prevailing features such as contrasting horizontal size of oceanic and land MCSs can be clearly detected by this study’s methods. In the next subsection, the covariability of cloud and precipitation is examined in detail using explicit correlation analysis.

### 3.2 Correlations between cloud and precipitation fractions

**Figure 8.**
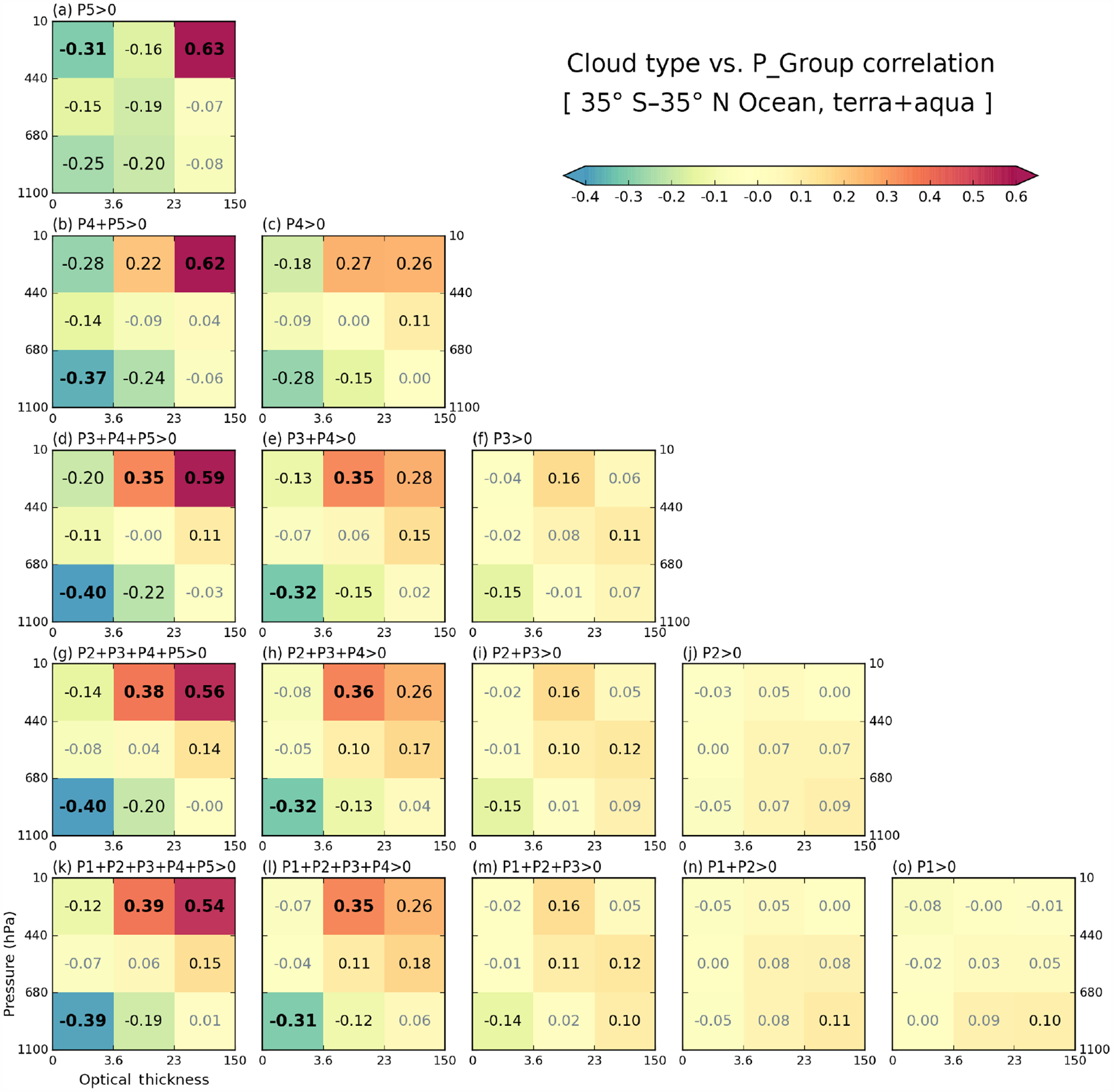
Cross-correlation coefficients in the extended tropical oceans for 13 years calculated between CFs of cloud types and precipitation group (individual or cumulative P-groups) values. The sum of all five precipitation groups shown in panel **(k)** corresponds to the total rainy fraction.

**Figure 9.**
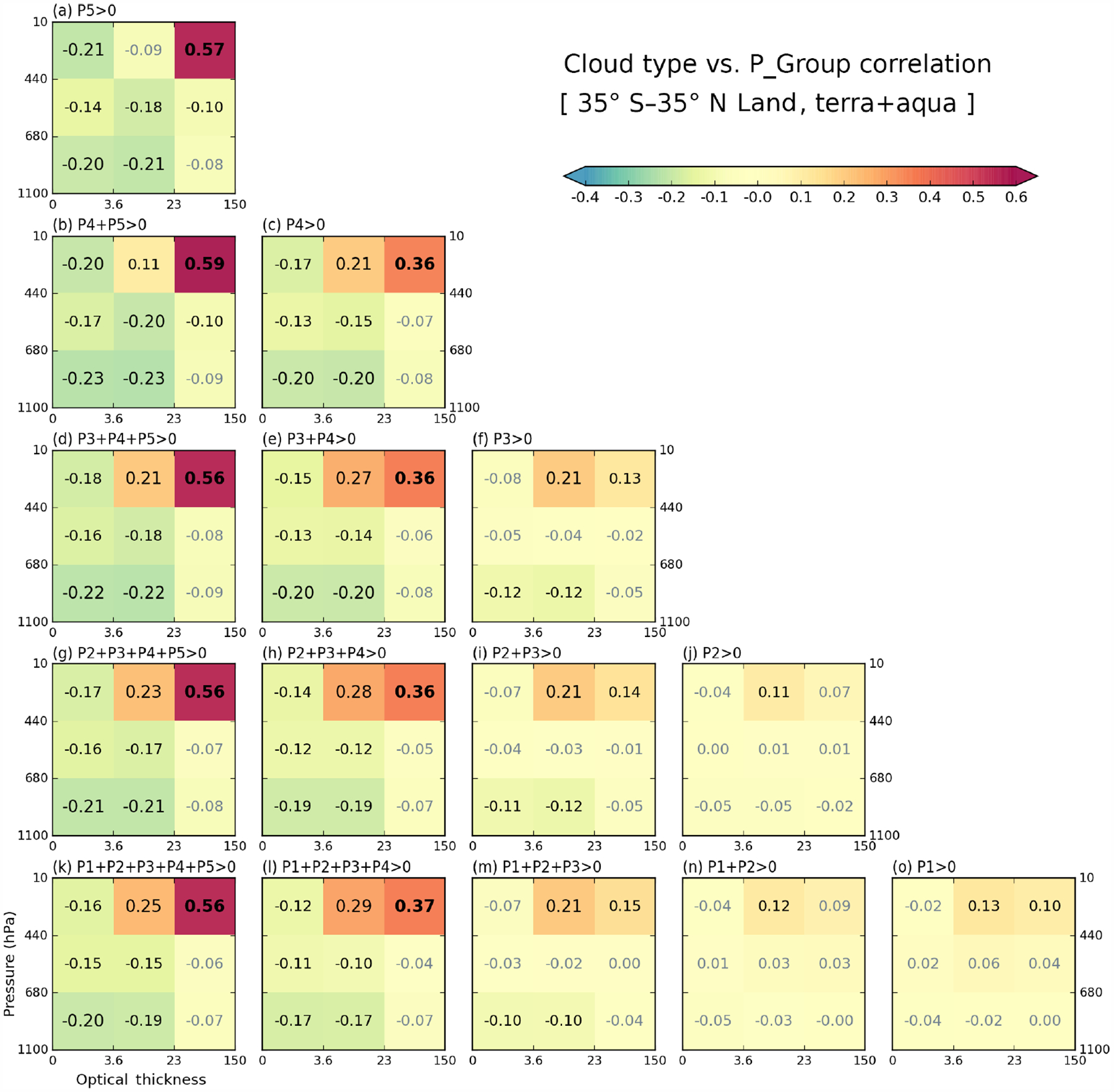
Same as Fig. 8, but over land.

As stated previously, to measure the co-variability of cloud and precipitation, we calculate cross-correlation coefficients between the CFs of the nine cloud types and the normalized frequencies (equivalent to fraction of precipitating area) within the five P-groups. Figures 8 and 9 show correlations of cloud types for each P-group as well as all combinations of consecutive cumulative P-group frequencies over the oceanic and land regions of our extended tropical domain. We note that when the fraction sum of specific P-group(s) is 0, the data point is excluded from the calculation of correlations. Hence, for example, correlation coefficients with P5 over ocean (Fig. 8a) are calculated with approximately 4.5 % of the total data available. Even over land, the sample size for this case (Fig. 9a) still exceeds 1 million, placing the 99 % significance level at less than 0.005 in terms of correlation coefficient absolute value. The statistical significance level was calculated here using a bootstrapping method which randomly shuffles the array, but in a way that considers the effect of autocorrelations between neighboring grid cells (i.e., shuffling by “chunks”; Kunsch, 1989; Léger et al., 1992; Liu and Singh, 1992). Consideration for the effect of neighboring grid cells is important because neighboring grid cells are usually *not* independent (e.g., a cloud system can occupy multiple grid cells); without this consideration, the degree of freedom will be overestimated, and thus the significance level underestimated. With the significance level quoted above, all correlations in Figs. 8 and 9 are statistically significant.

Examining first oceanic cloud-precipitation coupling, Fig. 8 reveals that strong correlations, both negative and positive, occur in the panels on the left, whilst correlations weaken as one moves to the right. The leftmost column panels consist of P-group(s) that include P5, the group of heaviest precipitation, while as one moves to the right, heavier precipitation is progressively excluded. The overall picture then is that of strong correlations corresponding mostly to heavy precipitation and of light precipitation correlating poorly with all cloud types. The leftmost column panels of Fig. 8 indicates that positive coefficients occur for high cloud types of moderate to strong optical thickness, namely cirrostratus (*Cs*; probably includes many anvils) and *Cb* (deep convection core), while negative values occur for low cloud types that are also optically thin. In the five panels of the leftmost column, *Cb* clouds always have strong positive correlations with precipitation, a result that comes as no surprise. For the correlation of *Cs* clouds to become positive and then increase, lighter precipitation has to be added to P5. For example, when only P5 values are used (Fig. 8a), the correlation coefficient of *Cs* clouds is negative (−0.16), but changes to 0.22 after P4 is added to P5 (Fig. 8b). This suggests that lighter rain in the vicinity of the heaviest rain is more closely related to *Cs* (or anvil) clouds. A similar trend of stronger correlations when lighter precipitation is added also ensues for low and thin *Cu* clouds, although with a negative sign in this case. In Fig. 8a, a strong negative correlation is seen with (high and thin) cirrus (*Ci*) clouds, and as lighter precipitation is added, the peak of negative correlations moves towards lower *Cu* clouds.

In order to get a sense of the physical reality represented by Pearson’s *r*, we examined two-dimensional histograms of cloud type CF and P-group for both strong positive and strong negative correlations (Figs. S2 and S3 in the Supplement). We note that more samples are available for 0 or small values of cloud type fraction for each case, and the distribution patterns look otherwise reasonable. We also examined the geographical dependence of these correlations and found them generally insensitive to location (Fig. S4 in the Supplement).

Notable patterns in correlation coefficients are also detected in the second left column panels which show correlations with P4 precipitation included, but without P5. Similar to the leftmost column panels, *Cb*, *Cs*, and *Cu* clouds show the stronger correlations with positive or negative signs. One difference from the P5 cases is that in Fig. 8e, h, and l, the positive correlations of *Cs* clouds are stronger than those of the thicker *Cb* clouds. The correlation coefficient values of *Cs* clouds in these panels are quite similar to the values for the same clouds in the leftmost column (which includes P5 precipitation). This result suggests that it is actually *Cs* clouds that are related the most to the variability of P4 and lighter precipitation.

Moving now to the land regions of our extended tropical domain, we use the same “correlation pyramid” to note that the relationship between high and optically thick *Cb* clouds and P5 heavy precipitation is of positive strength similar to that over oceans (Fig. 9). However, other details are quite different between land and ocean. First, the negative correlations of *Cu* clouds in the leftmost column panels are weaker. In Fig. 8, the peak negative correlation values reached −0.40 and occurred in panels (d) and (g), which include the moderate to weak precipitation of the P3 and P2 groups. In Fig. 9 on the other hand, the peak negative value weakens to −0.23 and occurs in panel (b), which represents the sum of only P4 and P5 precipitation; the negative correlations weaken as lighter precipitation is added. This result suggests greater chances of *Cu* clouds and P5 precipitation co-existing in 1°×1°grid cells over land compared to ocean. This observation may be related to our earlier finding inferred from Figs. 5–7 that the size of precipitating systems is much smaller over land than ocean.

Secondary but still noteworthy differences between land and ocean are identified in the correlations between *Cs* clouds and precipitation that include the P4 and P3 groups (second and third column panels from left in Figs. 8 and 9).Previously in Fig. 8, the maximum correlation values in the second-from-left column were the ones correlated with *Cs* clouds, up to 0.36. In the third column, associated with P3 precipitation, correlations with *Cs* clouds weaken to 0.16. In contrast, Fig. 9 shows that the strongest correlations of the second column are those for *Cb* clouds, not *Cs*. In the third column, the correlations with *Cs* clouds do not weaken as much, with a 0.21 correlation value being reached in P-groups that include P3. This pattern indicates that continental high clouds are more correlated with lighter precipitation. It is also notable that correlation coefficients with *Cs* clouds in the first column of Fig. 9 (including P5 over land) reach just 0.25, while those in Fig. 8 (ocean) are as high as 0.39. A possible explanation of the above correlation results is that thick anvils of continental MCS (Cetrone and Houze, 2009; Yuan et al., 2011) are more frequently classified as *Cb* rather than *Cs* (as defined in this study).

For light precipitation, the absolute values of correlation coefficients are smaller than those for heavy precipitation commonly found over land and ocean, reflecting the fact that the mechanisms and origins of light precipitation exhibit a greater variety. Nevertheless, a meaningful difference between land and ocean can be seen in Figs. 8 and 9. When comparing Fig. 8n and o with Fig. 9n and o, peak correlations around 0.1 occur for stratus (*St*) over ocean, but similar peak correlations over land occur even for *Cs* and *Cb*. This result suggests that over land even light precipitation is more frequently related to strong convective activity while oceanic light precipitation has a greater chance of being produced by “warm rain” mechanisms, as noted at the end of Sect. 3.1.

In summary, continental *Cs* and *Cb* clouds co-exist with a broader range of precipitation, but are also more weakly correlated with them, compared to their oceanic counterparts. This result is consistent with the previously noted climatological features of grid-mean cloud properties shown in Fig. 7. For example, the median *p*_*c*_ for the P2 group over land in Fig. 7d was already below 440 hPa, while for oceanic clouds the median *p*_*c*_ reached such values when precipitation was strong enough to belong to the P4 group. The optical thickness was also generally larger for land clouds (Fig. 7c). It is possible that TMPA is missing some “warm” rain events over land due to microwave retrieval inadequacies as stated in Sects. 2.1 and 3.1. For heavy precipitation, Level-2 TRMM observations led Liu et al. (2008) to conclude that tropical land storms are more vertically developed, i.e., with optically thicker clouds with higher tops, but also spatially more confined than oceanic storms (see also Houze et al., 2015; Matsui et al., 2016). Hence, precipitation over land occupies a smaller area, resulting in weaker correlations at scales of 1°. Differences in correlations between Figs. 8 and 9 therefore reflect land-ocean differences in the nature of tropical storms or MCSs.

There are other intriguing aspects of cloud-precipitation co-variability in land and ocean, and these are examined more closely in the next subsection: (1) the origin of negative correlations and (2) correlation sensitivity to precipitation strength.

### 3.3 Further investigation for correlation features

#### 3.3.1 Negative correlations between precipitation and thin clouds

**Figure 10.**
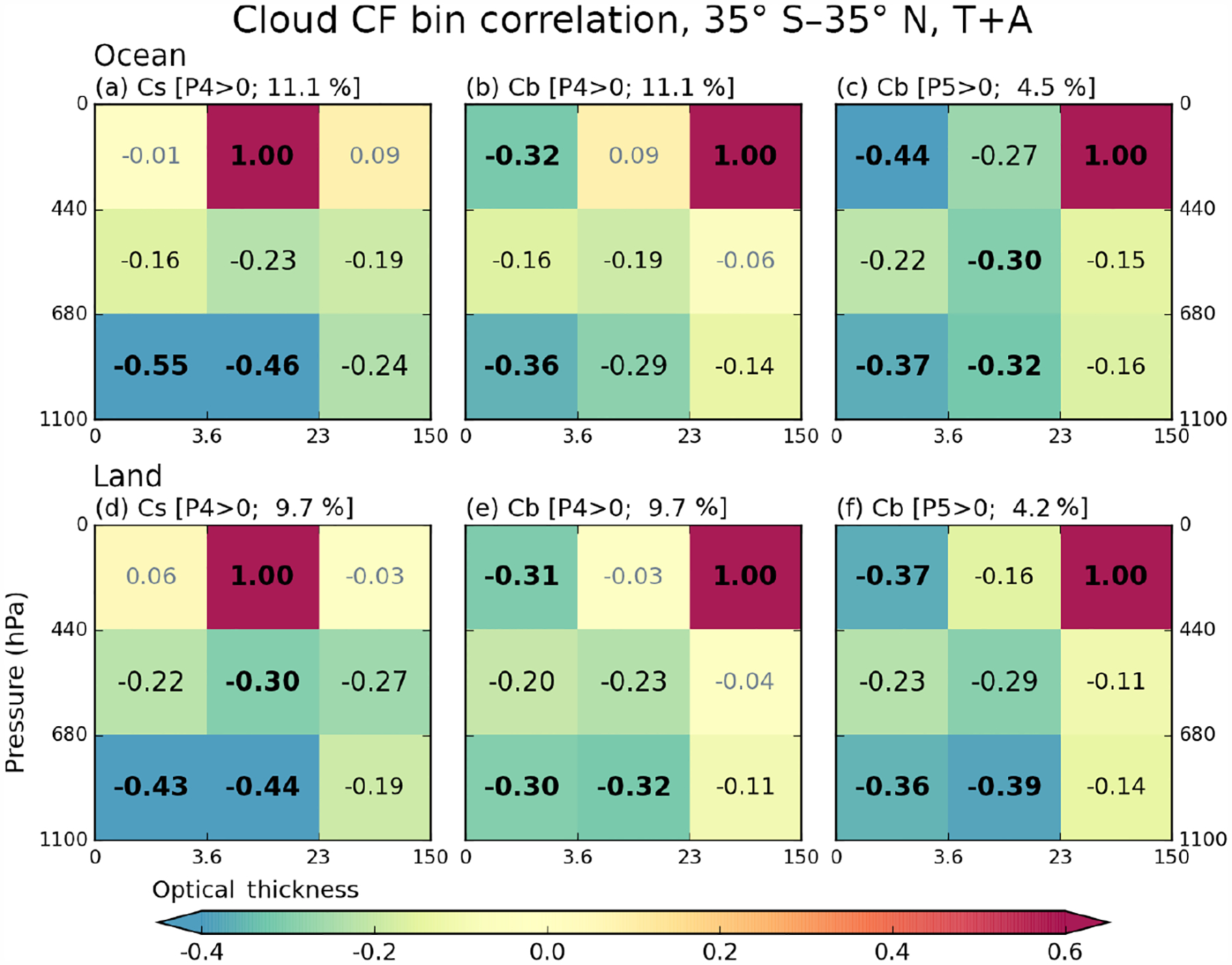
Conditional cross-correlation coefficients between cloud joint histogram bin CF values calculated for 13 years, based on **(a)** *Cs* CF over ocean when P4 *>* 0, **(b)** *Cb* CF over ocean when P4 *>* 0, **(c)** *Cb* CF over ocean when P5 *>* 0, **(d)** *Cs* CF over land when P4 *>* 0, **(e)** *Cb* CF over land when P4 *>* 0, and (f) *Cb* CF over land when P5 *>* 0. The percentage numbers above each panel are sample size ratios relative to the total number of ocean or land grid cells.

In Figs. 8 and 9, we saw thin clouds having negative correlations with heavy precipitation. These negative correlations can be interpreted as thin clouds being rarer when heavy precipitation occurs, an interpretation that is consistent with empirical observation and expectations. However, since it is also seen that heavy precipitation is strongly related to thick and high-level clouds (e.g., *Cb*), the negative correlation of optically thin clouds with heavy precipitation can also be interpreted as a contemporaneous negative co-occurrence relationship between optically thin low and optically thick high clouds. Please note that for a cloud type to be always (i.e., regardless of precipitation strength) anti-correlated with precipitation, its occurrence must be anti-correlated with that of other cloud types that are positively correlated with precipitation of a certain range. In order to examine these issues, we calculate internal correlations among cloud types based on the spatiotemporal variability of their CFs. From all possible combinations, we elect to show results where one of the cloud types is either *Cs* or *Cb* when P4 or P5 precipitation group occurs. These results for both land and ocean are shown in Fig. 10. For example, Fig. 10a (ocean) and d (land) show correlation coefficients between the CF of *Cs* and the CFs of all other cloud types for grid cells reporting P4 precipitation. We note that the samples used for Fig. 10 correlations are the same as those used for cloud–precipitation correlations shown in Figs. 8 and 9, given the same precipitation conditions, namely P4 or P5 values greater than 0 (i.e., Figs. 10a and b, and 8c).

Figure 10a, b, and c shows correlation coefficients based on oceanic *Cs* and *Cb* CFs. The *Cs* clouds are strongly anti-correlated with *Cu* and *Sc* clouds, while *Cb* clouds are furthermore strongly anti-correlated with *Ci* clouds. In the cases of P5 precipitation presence (Fig. 10c), the anti-correlation between *Cb* and *Ci* CFs becomes even stronger. Actually, in this case, *Cb* clouds are anti-correlated with all other cloud types; i.e., when *Cb* CF increases at 1°× 1°grid cell, CFs of other clouds decreases, and vice versa. These cloud type correlation patterns remind us of Fig. 8a, b, and c. For example, a comparison between Figs. 10c and 8a shows that the anti-correlation ordering by strength is the same, with *Ci* clouds coming first, *Cu* second, and *Sc* clouds third. This finding suggests that in tropical oceans P5 precipitation is mainly related to *Cb* clouds, and its anti-correlation with thin clouds is another expression of the anti-correlation between *Cb* and thin clouds. The exact nature of the anti-correlation is unknown because a passive sensor such as MODIS has limited skill in distinguishing between cases where the mid- and low-level clouds are absent and cases where they are obscured by high clouds.

When focus shifts to the weaker P4 precipitation group, both *Cs* and *Cb* clouds anti-correlate with low *Cu* and *Sc* clouds, and the anti-correlation is only slightly weaker for *Sc* than *Cu* (Fig. 10a and b). Previously however, Fig. 8c indicated that the anti-correlation between P4 group and *Sc* cloud is much weaker than that between P4 and *Cu* cloud (−0.15 vs. −0.28). This discrepancy is also seen in all panels of Fig. 8 representing correlations with moderate-to-heavy precipitation classes (third column from left), but is not seen over land (Fig. 9). While this issue will be discussed further in the next subsection, which deals with correlation sensitivity, suffice it to say here that cloud-precipitation anti-correlations cannot be exclusively attributed to cloud type co-occurrence anti-correlations.

When comparing oceanic and continental correlation patterns in Fig. 10 (top row vs. bottom row), we see the correlation patterns being quite similar, but with weaker correlation magnitudes over land. For example, *Cs* clouds in Fig. 10d remain strongly anti-correlated with *Cu* and *Sc* clouds, and *Cb* clouds in Fig. 10f are still anti-correlated with all other cloud types. However, differences between ocean and land clouds also emerge. First, particularly in the presence of nonzero P4 precipitation (Fig. 10d and e), there are stronger anti-correlations between *Cb* or *Cs* clouds and mid-level clouds over land. Previously in Fig. 6, we noted that mid-level clouds have greater CFs over land compared to ocean (even though their absolute value is much smaller than high clouds). The increased CFs of mid-level clouds over land may be related to a closer relationship with high thick clouds, thus affecting the correlation strength.

Another difference between ocean and land is the correlation between *Cb* and *Cs* in the presence of P5 precipitation. Comparing Fig. 10c and f, the notable anti-correlation value of −0.27 over ocean weakens to −0.16 over land. This result indicates that *Cb* and *Cs* clouds are less mutually exclusive over land. Since overcast conditions (100 % CF) in a 1°×1°grid cell are more frequent over ocean (Fig. 7b), in dicating that oceanic MCS can grow to sizes larger than 1°, there is a greater chance of competition between *Cb* and *Cs* over ocean to fill the grid cell.

Lastly, we return to our previous point that the anti-correlation of CFs among cloud types does not explain all features of the anti-correlation between cloud and precipitation shown in Figs. 8 and 9. For example, comparing Figs. 8a and 9a, anti-correlation between P5 and *Cu* cloud weakens from −0.25 (ocean) to −0.20 (land). However, Fig. 10c and f indicate that the anti-correlations between *Cb* and *Cu* clouds are almost the same for ocean and land (−0.37 vs. −0.36). This further supports the hypothesis that the frequencies of the P5 precipitation group and *Cb* CFs are more weakly coupled over land.

#### 3.3.2 Correlation sensitivity to heavy precipitation

**Figure 11.**
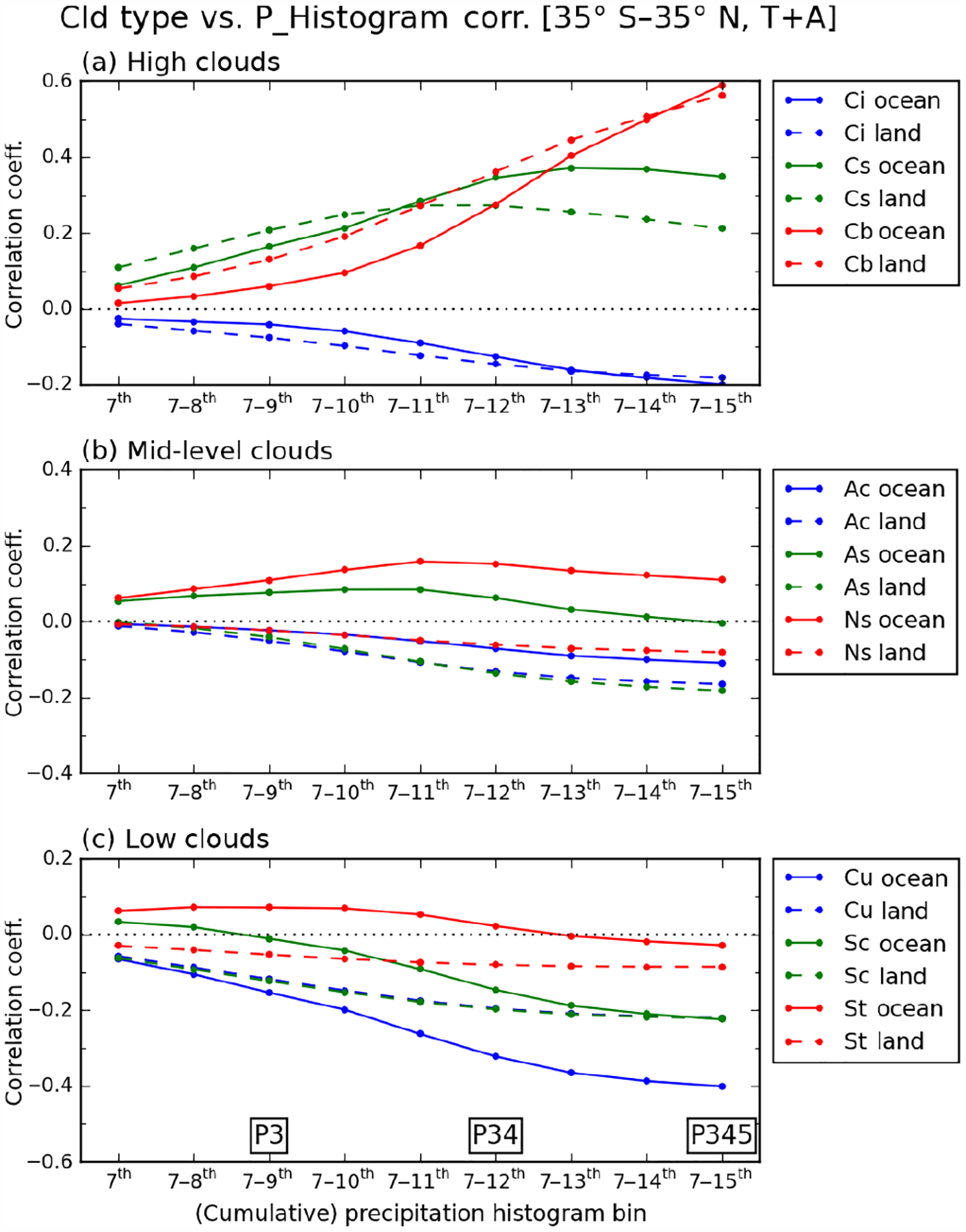
Correlation coefficients between cloud type CF and precipitation histogram values, for **(a)** high clouds (*Ci*, *Cs*, and *Cb*), **(b)** mid-level clouds (*Ac*, *As*, and *Ns*), and (c) low clouds (*Cu*, *Sc*, and *St*). Precipitation histogram values are added cumulatively from the seventh bin onward, so the sum from the seventh to the ninth bin corresponds to P3, and so on. Oceanic cloud results are shown in solid lines, and continental cloud results are shown in dashed lines.

Correlations between cloud and precipitation shown previously in Figs. 8 and 9 indicated that the heaviest precipitation group has a solid relationship (correlation or anti-correlation) with cloud types, while weaker precipitation groups do not. This fundamental finding is examined more closely with more detailed CF–precipitation correlations. Figure 11 shows correlation coefficients over both ocean and land between the CF of various cloud types and the frequency of cumulative precipitation within original precipitation bins, from the seventh bin onward (i.e., 0.251 mmh^−1^ and above). Hence, at the start of the *x* axis the precipitation frequency corresponds only to the seventh bin, and as one moves along the axis precipitation frequencies for subsequent bins are progressively added until the end of the axis where the precipitation frequency represents the sum of all values from the 7th to 15th bin, namely the sum of frequencies of the P3, P4, and P5 groups. When compared to Figs. 8 or 9, Fig. 11 shows essentially in more detail the evolution of correlation coefficients for the third row of the “pyramid”; i.e., correlation changes as one moves from Fig. 8f (9f) to 8e (9e), and then to 8d (9d) over ocean (land).

Figure 11a shows the correlation change of high clouds (*Ci*, *Cs*, and *Cb*). Over ocean (solid line), the correlation of *Cb* cloud increases monotonically as heavy precipitation is added, while that of *Cs* cloud peaks when the 13th bin (2.51–3.98 mmh^−1^) is added; further additions of heavier precipitation result in correlation coefficients trending downward. Similar patterns are also seen for the land clouds in this category. However, one prominent difference between ocean and land is that the land clouds in this group tend to be more strongly correlated with weaker precipitation. For example, continental *Cb* clouds correlate more than oceanic *Cb* with precipitation up to the 13th bin. However, the correlation curve for oceanic *Cb* clouds exhibits a steeper slope after adding the 11th bin, and ends up surpassing continental *Cb* clouds with the heaviest precipitation. In the case of *Cs* cloud, the continental correlation curve peaks with the addition of the 11th bin (1.0–1.58 mmh^−1^), while the oceanic peaks upon addition of the 13th bin. This result indicates that P4 precipitation over land tends to be more related with *Cb* than *Cs* clouds, contrasting what happens over ocean. In the case of *Ci* clouds, the anti-correlation is stronger at weak precipitation over land, consistent with the above argument, but the difference between land and ocean is not very pronounced given the small absolute values of coefficients compared to *Cs* and *Cb* clouds.

For the mid-level cloud group shown in Fig. 11b, a notable difference between ocean and land is seen for the *As* and *Ns* clouds. Oceanic *Ns* clouds have broad positive correlations around 0.1 for all precipitation bins. Oceanic *As* also have positive correlations with moderate-to-heavy precipitation bins, although they decrease to 0 as heaviest precipitation is added. On the other hand, continental *As* and *Ns* clouds show only negative correlations with all precipitation strengths. *As* and *Ns* occurrences are smaller over ocean (3.8, 1.7 %) than over land (5.3, 2.5 %) in Figs. 5 and 6 (when P4 *>* 0), but shallower convection over ocean seems sufficiently strong to produce moderate-to-heavy precipitation from *As* and *Ns* clouds.

In the case of the low cloud group shown in Fig. 11c, first, the thickest *St* clouds’ correlation evolution pattern looks similar to that of *As* clouds above, although the presence of *St* clouds over ocean is even smaller than *As* (*St* CF=1.3% vs. *As* CF = 3.8% when P4 *>* 0 in Fig. 5). The correlation pyramid of Fig. 8 showed that the positive correlation of *St* clouds is stronger when it is related to weak precipitation groups (P1 or P2), which are not included here (but are included in Fig. S5 in the Supplement). Secondly, also notable is the contrasting correlation evolutions of oceanic *Cu* and *Sc* clouds, previously mentioned to have different magnitudes of anti-correlation. Oceanic *Sc* clouds have slightly positive correlations with the seventh and seventh to eighth precipitation bins, which then become negative as heavier precipitation is added. Similar to the *St* cloud, the positive correlation of *Sc* cloud is expected to strengthen with even lighter precipitation (Figs. 8 and S5 in the Supplement). For the oceanic shallow convection, our results of low- and mid-level cloud correlations consistently indicate that shallower and thinner clouds (e.g., *Sc*) are more related to lighter precipitation, while higher and thicker clouds (e.g., *Ns*) are more related to heavier precipitation. In the case of *Cu*, the correlation coefficient is roughly the same between ocean and land for the seventh precipitation bin, but the correlation curves diverge as heavier precipitation is added. By the time the frequencies of all precipitation bins from 7th to 15th have been added, oceanic *Cu* clouds have twice as strong anti-correlation compared to their continental counterparts. As discussed previously in the context of Fig. 10, correlations among cloud fraction co-occurrence, i.e., *Cu* vs. *Cs* or *Cu* vs. *Cb*, are not as different between ocean and land as those shown here between clouds and precipitation. The weaker anti-correlation of continental *Cu* cloud with rainfall reflects then, at least partly, the less robust relationship between heavy precipitation and continental high clouds.

### 3.4 Limiting factors and uncertainties

#### 3.4.1 Uncertainty of cloud type classification

In this study, MODIS-observed clouds are classified into nine cloud types adopted from previous ISCCP conventions (Chen et al., 2000; Rossow and Schiffer, 1999) for the sake of convenience. This classification is, strictly speaking, based on arbitrary *τ* and *p*_*c*_ thresholds, and clouds assigned to each pair of bin boundaries will only loosely represent cloud types originally defined from morphological features seen by surface observers. Previously we noted that continental MCSs often include thick anvils (Cetrone and Houze, 2009; Yuan et al., 2011), but we can not confirm that these anvils are classified as *Cs* or *Cb* without knowledge of the cloud vertical extinction profile. Moreover, a passive sensor like MODIS has intrinsic limitations in identifying certain cloud types. Recent studies examining the nature of MODIS cloud regimes with active sensor observations from CloudSat and the Cloud-Aerosol Lidar and Infrared Pathfinder Satellite Observations (CALIPSO) show that similar MODIS joint histograms can have a variety of cloud vertical structures (Oreopoulos et al., 2017). In addition, Wang et al. (2016) showed that defining cloud types from CloudSat-CALIPSO observations where cloud vertical extent is better known can yield large disagreements with cloud type definitions from the MODIS *p*_*c*_–*τ* joint histogram. Such ambiguous definitions of cloud types from passive measurements may be the reason for substantial correlations between *As* and certain ranges of precipitation even though *As* is usually thought of as a non-precipitating cloud type. In summary, the nine cloud types in this study may not strictly correspond to their traditional, ground-based classification, so their relationship with precipitation should not be taken literally or juxtaposed with empirical knowledge. They are simply a convenient framework to organize findings about cloud–precipitation co-variability at 1°scales.

Furthermore, the passive MODIS observations suffer from low skill in detecting multi-layer clouds. Specifically, MODIS generally only detects the cloud top of the highest cloud, so high clouds such as cirrus or stratiform anvil will mask the presence of shallow clouds. This may be a contributing factor to the negative correlations by *Cu* and *Cs* in Fig. 10. Unfortunately, this is a shortcoming of passive cloud observations that we have to live with in exchange for wider coverage.

#### 3.4.2 Uncertainty of TMPA and its temporal matching to MODIS

As noted in Sect. 2.1, TMPA quality varies by location. Over land, the strong surface emissivity forces microwave retrievals of precipitation to rely on the ice scattering signature, which may not be present for warm (or shallow) rain. While there are gauge adjustments over land, they depend on the quality and density of the gauges used and operate at monthly timescales; thus they may not be able to correct the precipitation rates for individual rain events. Over ocean, gauge adjustment is unavailable, leading to potential systematic errors in the precipitation estimates. Furthermore, the retrieval of remotely sensed precipitation relies on algorithms that estimate surface precipitation rates from passive microwave brightness temperature, a task that remains challenging. In addition, due to the intermittency of passive microwave sensors on low-Earth orbit satellites, gaps in the microwave field are filled in by infrared-based precipitation estimates, which have poor accuracy as infrared brightness temperature in isolation is only indirectly related to precipitation (it is as if we were trying to correlate precipitation here with one-dimensional *p*_*c*_ histograms). Hence, TMPA estimates possess considerable uncertainties.

Furthermore, precipitating systems can develop quickly, especially over land. For example, a tropical squall line can develop in a few hours, so it is possible that MODIS and TMPA observe different stages of a system given that a 1.5 h difference is possible in spite of our temporally matching. This situation can result in decreased correlation coefficients between high thick clouds and heavy precipitation over land. We are somewhat less concerned about this sampling issue because the lead/lag time between MODIS and TMPA is expected to be random, and therefore hopefully not a source of systematic bias. In the future, this concern can be ameliorated by using a higher temporal resolution precipitation data set such as the Integrated Multi-satellitE Retrievals for GPM (IMERG; Huffman et al., 2018) instead of TMPA.

## 4 Summary and conclusion

The total amount, intensity, and frequency of precipitation should be organically related to the properties of the clouds from which they originate. However, due to the different radiative signal strengths of hydrometeors at particular parts of the electromagnetic spectrum, precipitation and cloud observations are significantly decoupled, necessitating joint analysis of products developed for different purposes and from imperfectly matched observations. Even with such non-ideal data at hand, the community still aspires to answer fundamental questions such as the following: to what degree can precipitation be predicted given information about clouds? Conversely, with precipitation information at hand, can we provide good guesses about the nature of the clouds responsible? Is precipitation variability associated with cloud variability? Do answers to the above questions differ substantially between ocean and land? This paper seeks to contribute ideas and results that will help us make progress in obtaining concrete answers in the near future, especially if observations also make considerable strides.

In order to work towards solving the problem of understanding cloud-precipitation co-variability, we use contemporaneous multi-year data sets, widely accepted concepts about how to classify clouds into various types from passive observations, and a combination of compositing and correlation analysis. We try to preserve some sub-grid variability information at 1°scales by employing precipitation histograms built from the TMPA data set, as well as MODIS joint histograms of cloud top pressure and cloud optical thickness, both of which are matched spatiotemporally.

We find, not surprisingly, that correlations between deep convective clouds and heavy rainfall are strong and stand out clearly, dwarfing all other correlation combinations for both land and ocean. Land-ocean differences are also remarkable. For example, oceanic deep convection systems (e.g., mesoscale convective systems) are more likely to attain overcast conditions and to have larger fractions of rainy sub-grids within 1°× 1°grid cells, both indicative of larger horizontal size than their continental counterparts, consistent with previous studies. Over land on the other hand, *Cb* and *Cs* clouds are related not only with heavy precipitation, but rather with a broader range of rainfall which translates to weaker correlations.

Thin clouds, particularly *Cu* clouds (as defined here) are anti-correlated with moderate-to-heavy precipitation. The anti-correlation is stronger over ocean, and the magnitude is comparable to the anti-correlation between *Cu* and high thick clouds (*Cb* or *Cs*). The fact that oceanic deep convection often fills and outgrows the 1°× 1°reference grid cell, is ultimately the cause of clearer relationships (less uncertainty) among heavy precipitation, high thick clouds, and low thin clouds.

Over ocean, low- and mid-level clouds also exhibit positive correlations with precipitation of certain ranges, which represents shallow convection and warm rain processes. Among those clouds, the relatively higher and thicker *Ns* clouds are more related to moderate-to-heavy precipitation, while lower and thinner *Sc* clouds are more related to light precipitation. In the end, positive correlations indicate that oceanic precipitation comes from a variety of cloud types and rain formation processes (warm rain) while most precipitation over land requires the presence of high clouds. Notably, the shallow continental clouds show stronger anti-correlations with heavy precipitation than positive correlations with light precipitation. It is conceivable that this result can change once detection of low clouds in the presence of high clouds and of warm rain over land improves (Field and Heymsfield, 2015; Mülmenstädt et al., 2015).

Collectively, we make a strong case that rainfall predictability is better over oceans than continents when cloud information is available. But even over oceans, there are significant uncertainties in linking certain ranges of precipitation with specific cloud types, at least with our approach. Our self-imposed objective to make the study general, multi-year, and applicable to half of the Earth’s surface led us to Level-3 gridded data as the most appropriate choice. While some of the details seen in previous studies that used Level-2 data will unavoidably be lost, our data sets are good enough to extract major features of cloud–precipitation co-variability and allow us to claim that they are broadly representative of this co-variability in the tropics. We argue that the insensitivity of cloud-precipitation relationships to location (Fig. S4 in the Supplement) and precipitation data set (initial tests with recent GPM-IMERG data that may be presented in a future study yielded similar results) strengthens the validity of this conclusion.

We expect that our study has the potential to form the basis for enhanced evaluation of precipitation in GCMs. A regime-based analysis in the deep tropics by Tan et al. (2017) suggests that clouds and precipitation are more decoupled in models than in observations (see also Jing et al., 2017; Suzuki et al., 2015). Confirming that conclusion with the approach introduced in this study is a possible next endeavor. In addition, more effort should be extended to apply the framework in this study to various case studies with more appropriate data sets (e.g., using higher-resolution precipitation data sets for regional/seasonal studies, or longer period data sets for climate studies) in order to increase further our degree of confidence about the cloud-rainfall relationships.

## Supplementary Material

Supplement

## Figures and Tables

**Table 1.** Population percentages of grid cells with specific precipitation characteristics over ocean and land from 13 years of data in our 35°S–35°N extended tropics domain.

	Ocean	Land
P0> 0.5	85.21%	89.95%
P4> 0	11.13%	9.73%
P5> 0	4.52%	4.23%
P4+P5> 0	11.41%	10.13%
P4> 0 and P5> 0	4.27%	3.83%
