# Supplementary material for: Contrasting the co-variability of daytime cloud and precipitation over tropical land and ocean
Source: Atmos Chem Phys. Author manuscript; Available in PMC 2020 Jul 13. (PMC7356930; doi:10.5194/acp-18-3065-2018)
Supplement: Supplement [file NIHMS1502892-supplement-Supplement.pdf]

Supplement of Atmos. Chem. Phys., 18, 3065–3082, 2018
https://doi.org/10.5194/acp-18-3065-2018-supplement
© Author(s) 2018. This work is distributed under
the Creative Commons Attribution 4.0 License.

Atmospheric Chemistry and Physics
Open Access
EGU

Supplement of

Contrasting the co-variability of daytime cloud and precipitation over tropical land and ocean

Daeho Jin et al.

Correspondence to: Daeho Jin (daeho.jin@nasa.gov)

The copyright of individual parts of the supplement might differ from the CC BY 4.0 License.

Supplementary Figures

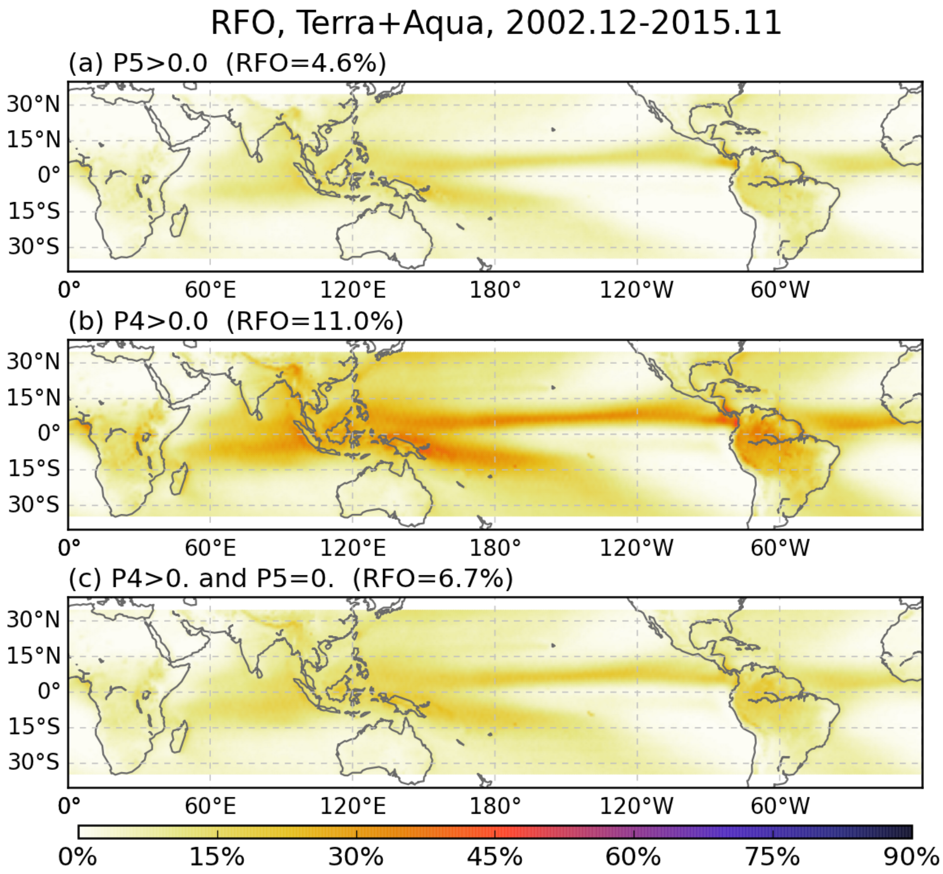

Supplementary Figure 1: Map of relative frequency of occurrence (RFO) of the following conditions: (a) P5 values greater than 0; (b) P4 values greater than 0; and (c) P4 greater than zero, but with P5=0.

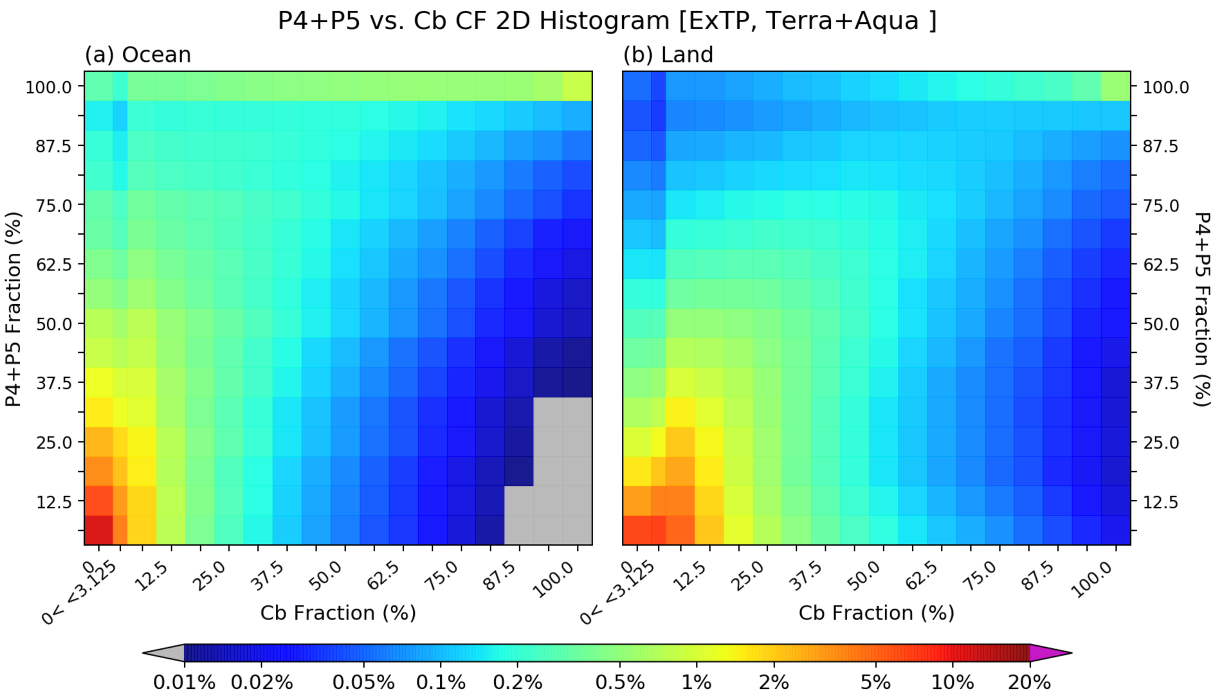

Supplementary Figure 2: 2D joint histograms of P4+P5 fraction and Cb cloud fraction for (a) ocean and (b) land regions. As Figs. 8 and 9, the samples in the calculation are conditional to P4+P5 > 0. The histogram values are normalized to represent percentage of total number of samples. Gray color indicates values below 0.01%, and white color indicates 0% (no sample). The histogram bin size is 1/16 (=6.25%), and the bin labeled as "50%" indicates bin boundaries from 46.875% to 53.125%.

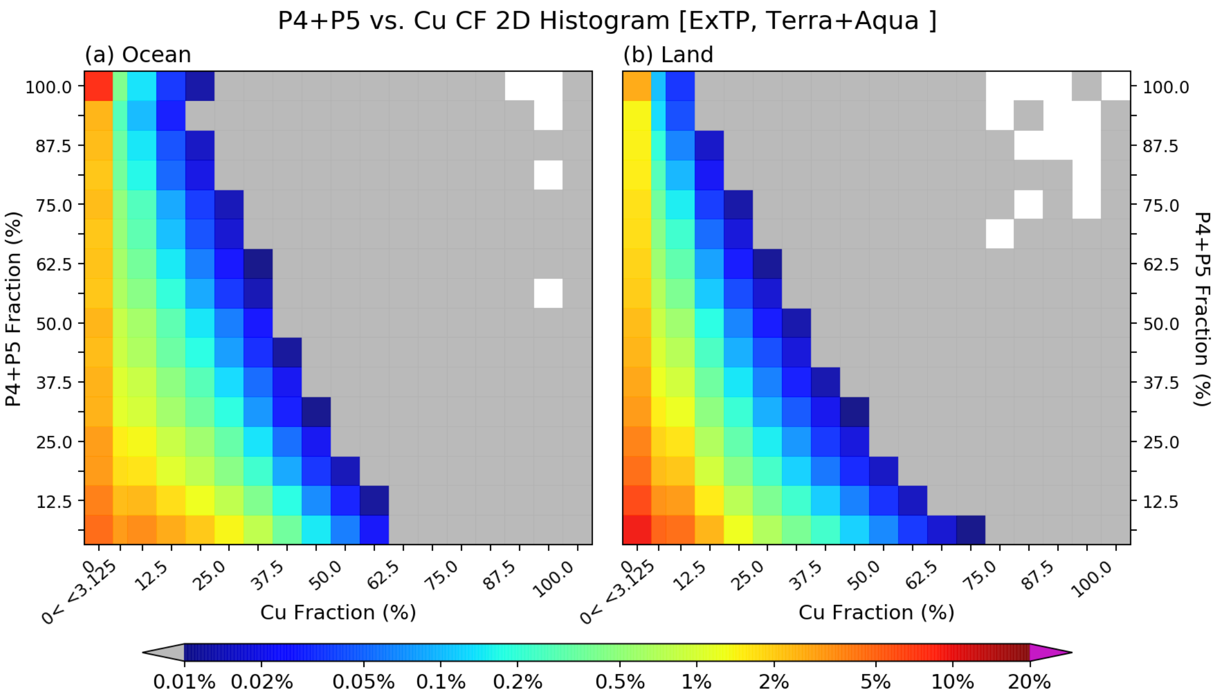

Supplementary Figure 3: Same as Supplementary Fig. 2, but for Cu cloud.

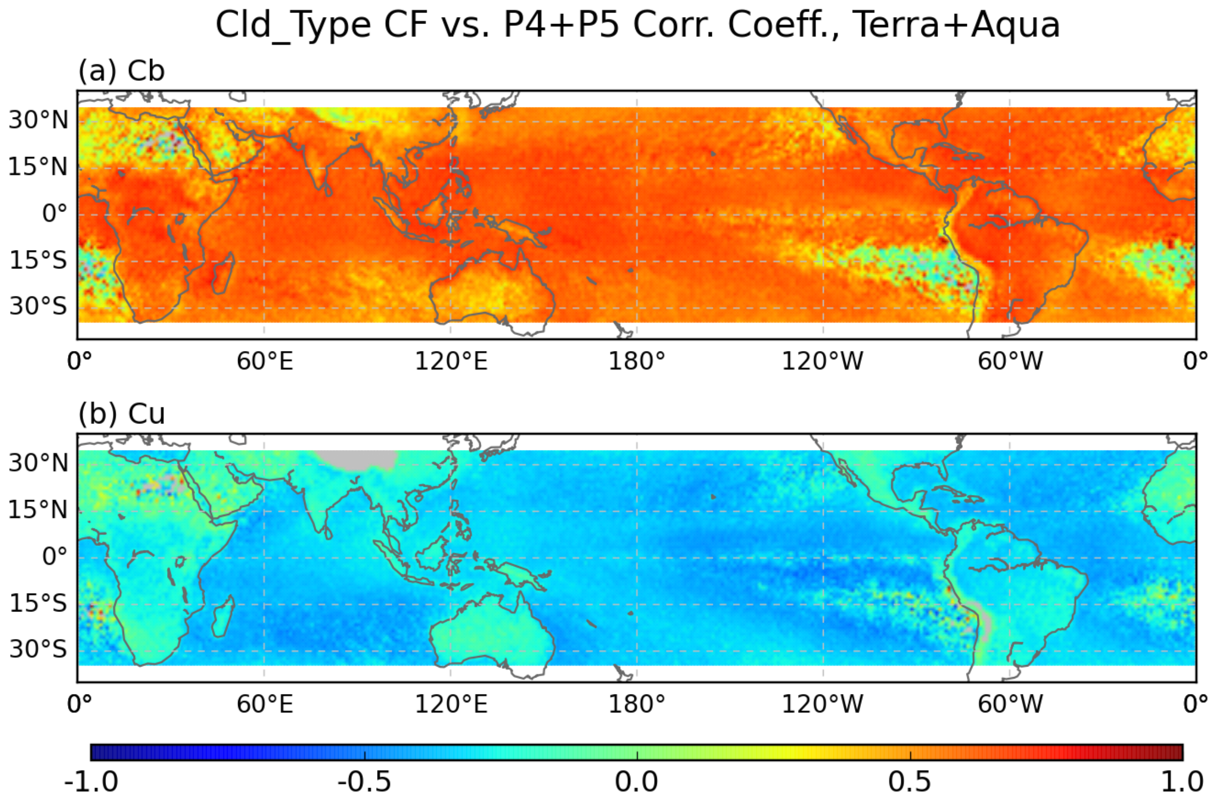

Supplementary Figure 4: 1°×1° resolution maps of correlation coefficients between (a) Cb or (b) Cu cloud fraction and P-group fraction P4+P5. As in Figs. 8 and 9, the samples for the calculation are conditional to P4+P5 > 0. The regions of abnormally high or low correlation values (e.g., Sc-dominant regions, the Sahara, the Himalayas, etc.) have usually small sample sizes.

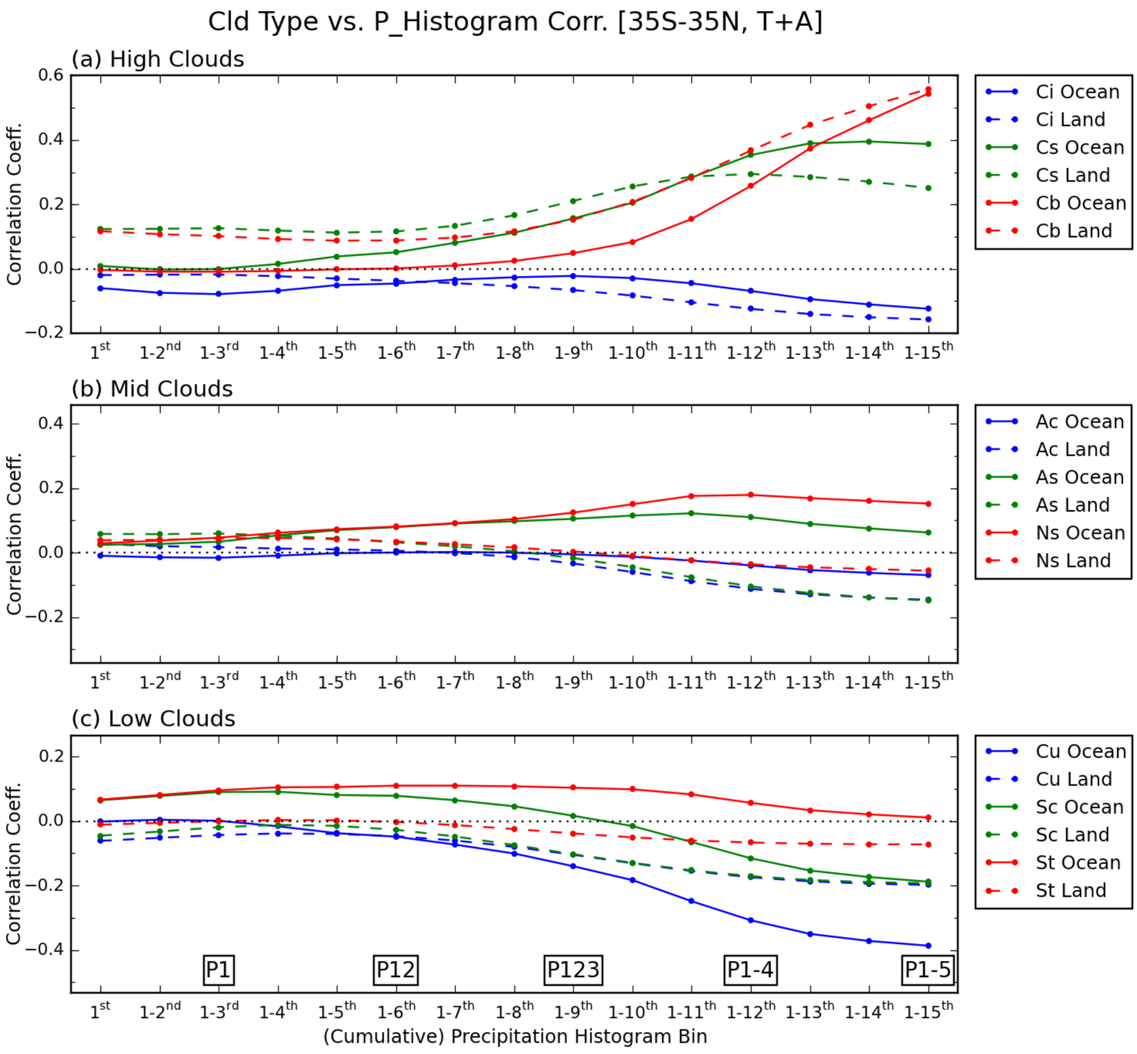

Supplementary Figure 5: Same as Fig. 11, but with the x-axis starting at the 1st precipitation bin and cumulatively adding up to the heaviest 15th bin, which is equivalent to the sum from P1 to P5 or total rainy fraction. This figure therefore shows in more detail the evolution of the correlation coefficients in the panels of the bottom row of Figs 8 and 9.

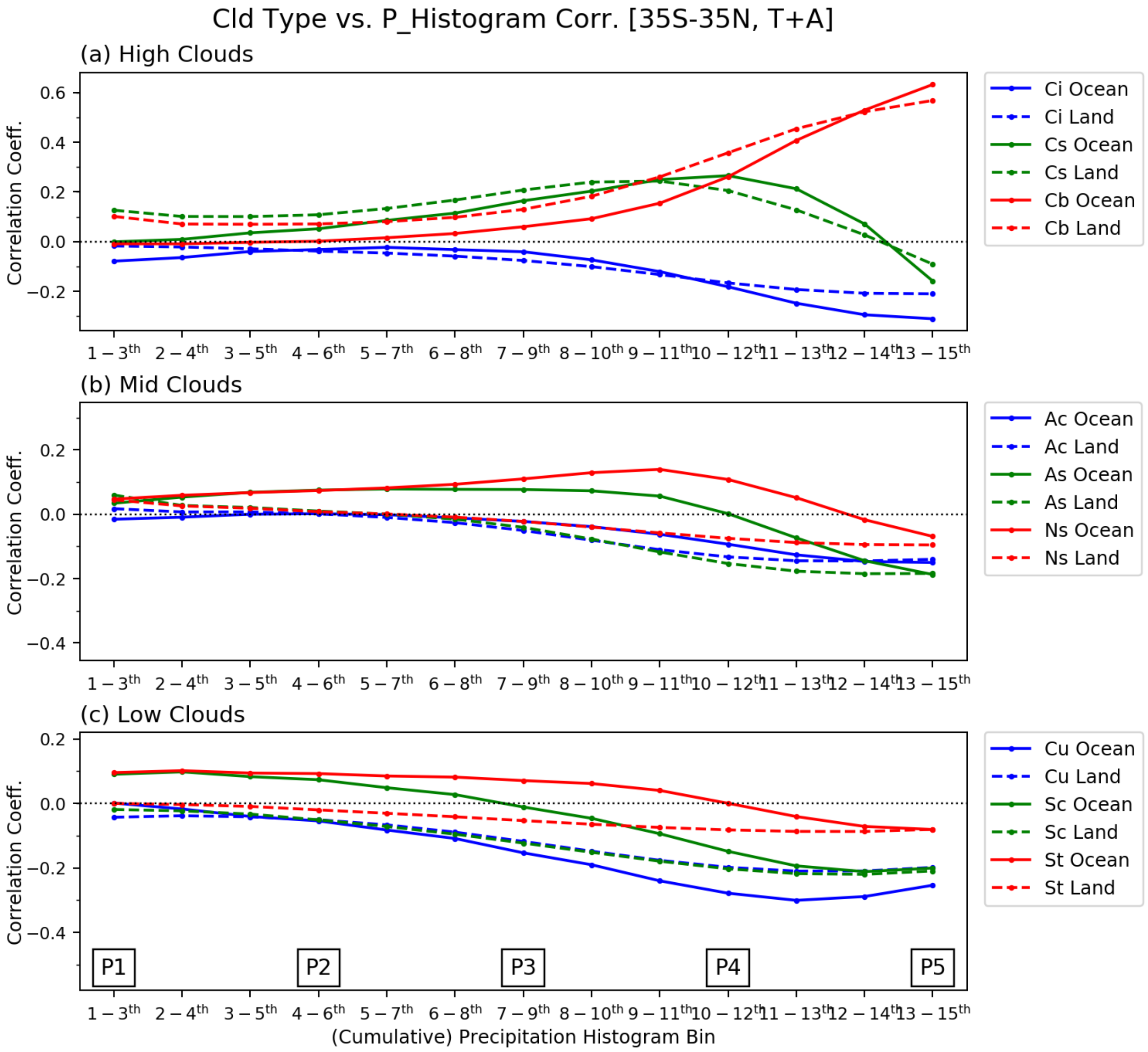

Supplementary Figure 6: Same as Fig. 11, but with the x-axis starting at the combined 1st-3rd histogram bins (P1), and then progressing as a running sum of three consecutive histogram bins up to the rightmost point corresponding to P5.
